# Focal astrocyte loss reveals nuclear translocation during lesion repopulation

**DOI:** 10.1038/s41593-026-02354-5

**Published:** 2026-07-23

**Authors:** Marina Herwerth, Matthias T. Wyss, Nicola B. Schmid, Anna Lasne, Jacqueline Condrau, Luca Ravotto, José María Mateos Melero, Andres Kaech, Gustav Bredell, Carolina Thomas, Rachel Kim, Petra Kukanja, Vladyslav L. Korobeynyk, Christine Stadelmann, Thomas Misgeld, Jeffrey L. Bennett, Sebastian Jessberger, Aiman S. Saab, Shane A. Liddelow, Bruno Weber

**Affiliations:** 1 Institute of Pharmacology and Toxicology, University of Zurich, Zurich, Switzerland; 2 Neurology Department, University Hospital Zurich, University of Zurich, Zurich, Switzerland; 3 Neuroscience Center Zurich, University and ETH Zurich, Zurich, Switzerland; 4 Institute of Neuronal Cell Biology, Technical University of Munich, Munich, Germany; 5 Department of Neurology, Klinikum rechts der Isar, Technical University of Munich, Munich, Germany; 6 Center for Microscopy and Image Analysis, University of Zurich, Zurich, Switzerland; 7 Computer Vision Laboratory, Department of Information Technology and Electrical Engineering, ETH Zurich, Zurich, Switzerland; 8 Department of Neuropathology, University Medical Center Göttingen, Göttingen, Germany; 9 Cluster of Excellence ‘Multiscale Bioimaging: from Molecular Machines to Network of Excitable Cells’ (MBExC), University of Goettingen, Göttingen, Germany; 10 Institute for Translational Neuroscience, NYU Grossman School of Medicine, New York City, NY USA; 11 Brain Research Institute, University of Zurich, Zurich, Switzerland; 12 German Center for Neurodegenerative Diseases (DZNE), Munich, Germany; 13 Munich Cluster for Systems Neurology (SyNergy), Munich, Germany; 14 Departments of Neurology and Ophthalmology, Programs in Neuroscience and Immunology, University of Colorado School of Medicine, Aurora, CO USA; 15 Department of Neuroscience, NYU Grossman School of Medicine, New York City, NY USA; 16 Department of Ophthalmology, NYU Grossman School of Medicine, New York City, NY USA; 17 Parekh Center for Interdisciplinary Neurology, NYU Grossman School of Medicine, New York City, NY USA

**Keywords:** Astrocyte, Neuroimmunology

## Abstract

Astrocyte loss occurs in various neurological conditions and can disrupt local tissue homeostasis. While astrocytes surrounding border-forming lesions adopt reactive states without restoring astrocyte networks, how astrocytes respond to spatially confined astrocyte loss remains poorly understood. Here we used longitudinal in vivo two-photon microscopy, combined with spatiotemporal transcriptional profiling, to examine astrocyte responses following focal aquaporin-4 antibody-mediated ablation in the somatosensory cortex of adult mouse brain, a model of astrocytopathy relevant to neuromyelitis optica spectrum disorder. Here we show that perilesional astrocytes undergo pronounced structural remodeling during lesion repopulation, characterized by cell proliferation, prolonged multinucleated astrocyte states, polarized process extension into the depleted area and gradual displacement of nuclei into previously unoccupied astrocyte territories. Spatial transcriptomics reveal an injury-associated molecular response that resolves as the astrocyte network is restored. Together, our findings delineate the spatiotemporal dynamics of astrocyte regeneration after astrocyte loss, extending current understanding of astroglial plasticity in the adult brain.

## Abstract (web summary)

Herwerth et al. demonstrate that, after focal astrocyte loss in the adult brain, perilesional astrocytes undergo extensive remodeling, proliferation and nuclear translocation to repopulate the depleted tissue without forming a glial scar.

## Main

In response to various types of brain injury^1–3^, astrocytes undergo a process called reactive astrogliosis. This action encompasses coordinated morphological, molecular and functional changes^4–6^, and has been studied in detail in traumatic central nervous system (CNS) injury models. Under these circumstances, reactive astrocytes remain localized to the lesion boundary, upregulate several neural precursor cell (NPC) hallmark proteins and form a glial border^7,8^. A fraction of these cells becomes multinucleated and undergoes limited symmetric proliferation, typically one division and rarely two divisions after repetitive traumatic brain injury^9^. The daughter cells remain stationary in close vicinity to each other and stay within the astrocyte lineage^9,10^. In brain pathologies involving vessel disruption, these cells are restricted to the juxtavascular niche^9^, suggesting that blood–brain barrier dysfunction is a trigger for astrocyte proliferation^11^.

Although reactive astrogliosis is heterogeneous across different stimuli, key features include cellular hypertrophy, upregulation of glial fibrillary acidic protein (GFAP), limited proliferation and activation of distinct transcriptional programs^4^. Previous studies suggest that the context-dependent remodeling of reactive astrocytes can promote many different responses, including neuroinflammatory, neurodegenerative, proliferative and regenerative—each with different context-dependent outcomes for the surrounding neural tissue^4,12^.

Despite proliferative activity, astrocytes fail to repopulate border-forming CNS lesions; instead, they try to seal the necrotic CNS tissue by forming a tight surrounding border. The glial border itself is at least in part a dysfunctional structure, and its contribution to regeneration remains a topic of debate^8,13^. This raises the question of whether astrocytes in the adult brain have intrinsically lost their regenerative capacity, or whether border-containing lesions limit their regenerative potential. However, current understanding of astrocytic remodeling upon injury in the adult brain in vivo is primarily based on the models involving direct mechanical, hypoxic or global tissue damage^4^. In contrast, in autoimmune diseases such as neuromyelitis optica spectrum disorder (NMOSD), astrocytes are selectively attacked and depleted^14,15^. How surviving astrocytes at the lesion margins respond, remodel and contribute to tissue regeneration in this context remains poorly studied.

Here we used an antibody-mediated strategy to ablate astrocytes in a selective and locally controlled manner in the otherwise intact adult brain. We targeted the water channel aquaporin-4 (AQP4), which is almost exclusively expressed by astrocytes in the CNS^16^. Autoantibodies against AQP4 can be found in patients with NMOSD—a paradigmatic autoimmune disease of the CNS with B-cell-orchestrated immune responses^14,17^. By tracking perilesional astrocytes with chronic, intravital two-photon microscopy in anesthetized and awake mice, we discover that mature cortical astrocytes undergo a remarkable remodeling, including the translocation of adult-born daughter-cell nuclei to newly unoccupied astrocytic territories, resulting in complete and rapid repopulation of the astrocyte-depleted area within a few weeks. This remodeling process is associated with a distinct injury-related transcriptomic signature and is neither associated with the vascular niche nor dependent on connexin 30/connexin 43 (Cx43) signaling. This repopulation behavior is limited in focal stroke lesions of comparable volume. Applying an alternative all-optical apoptotic ablation method to astrocytes^18^, we identify the extent of astrocyte loss—suggesting disruption of contact-inhibitory signaling—as a potential modulator of the observed astrocyte remodeling response. Notably, comparable astrocytic features are also apparent in AQP4-IgG-mediated lesions from patients with NMOSD. We propose that, in response to local astrocyte loss, mature astrocytes undergo highly dynamic transcriptional and structural remodeling that is associated with the re-establishment of astrocytic tiling.

## Results

### Astrocytes repopulate rapidly after focal astrocyte loss

**Fig. 1:**
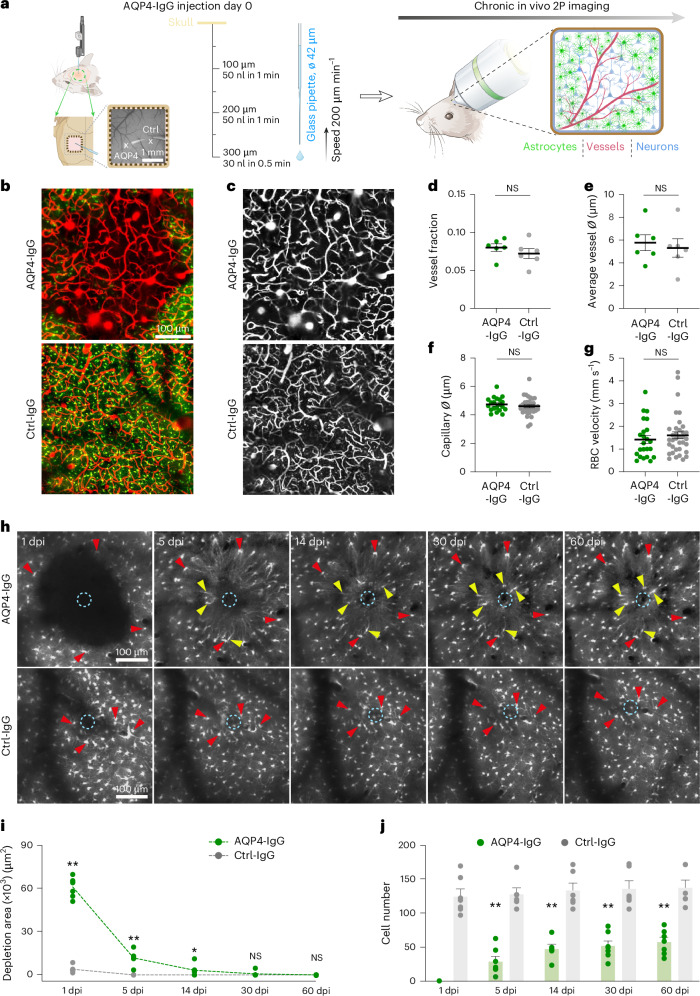
AQP4-IgG-based selective astrocyte depletion leads to efficient astrocytic lesion repopulation. **a**, Protocol of AQP4-IgG-based astrocyte depletion in the somatosensory cortex of anesthetized mice. Implantation of a permanent window allows chronic in vivo two-photon microscopy imaging. **b**, Projected two-photon image *z* stacks (100 µm) from *Aldh1l1*^GFP^ mice, demonstrating focal loss of astrocyte-expressing GFP signal. Vessels were visualized with administered intravenous injection of Texas Red dextran (70 kD). **c**, *Z* projections of the vascular channel from **b** (same scale as in **b**). **d**–**g**, AQP4-IgG-mediated ablation of astrocytes does not alter vascular structure compared to the intracortical injection of a control antibody (Ctrl-IgG). Vessel fractions (*n* = 6 animals per condition; **d**) and average vessel diameters (*n* = 6 animals per condition; **e**) determined in vessel segmentations (Mann–Whitney test, two sided). Average capillary diameters (AQP4-IgG—*n* = 20 capillaries from three animals; Ctrl-IgG—*n* = 35 capillaries from three animals; **f**) and RBC velocities in capillaries measured in vivo (AQP4-IgG—*n* = 20 capillaries from three animals; Ctrl-IgG—*n* = 35 capillaries from three animals; Mann–Whitney test, two sided; **g**). **h**, Using longitudinal in vivo two-photon imaging in *Aldh1l1*^GFP^ mice, recoverage of the astrocyte-depleted area after AQP4-IgG injection was followed over 60 days (top). Cells in the direct vicinity of the lesion (perilesional astrocytes, red arrowheads) remain in their location throughout the experimental time period. Within the initially astrocyte-depleted area, an increasing number of newly appearing astrocytes are detected (yellow arrowheads) over time. Bottom: after Ctrl-IgG injection, cells in the direct vicinity of the injection site (cyan dashed circles) are present throughout the whole follow-up period (red arrowheads). **i**,**j**, Quantification of mean depletion area size over time (AQP4-IgG, 1 dpi, 14 dpi, 30 dpi and 60 dpi—*n* = 7 lesions from six animals; 5 dpi—*n* = 5 lesions from four animals; Ctrl-IgG, 1 dpi, 14 dpi, 30 dpi and 60 dpi—*n* = 6 lesions from six animals, 5 dpi—*n* = 5 lesions from five animals) in **i** and the cell number in the lesion area in **j** (AQP4-IgG, 1 dpi—*n* = 9 lesions from eight animals, 5–60 dpi—*n* = 7 lesions from six animals; Ctrl-IgG, 1 dpi—*n* = 7 lesions from seven animals, 5–60 dpi—*n* = 6 lesions from six animals). *P = 0.021, **P < 0.01. (Mann–Whitney test, two sided). Data are represented as mean ± s.e.m. RBC, red blood cell; NS, not significant. Schematic in **a** partly created in BioRender; Wyss, M. https://BioRender.com/xib2ya2 (2026). Source data

**Extended Data Fig. 1:**
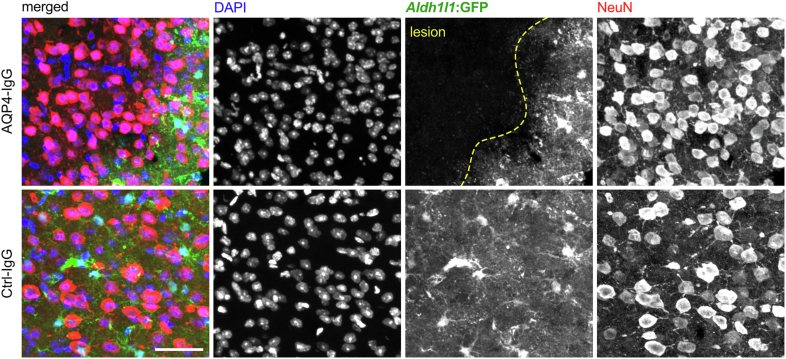
AQP4-IgG injection selectively depletes astrocytes. Confocal images of endogenous *Aldh1l1*^GFP^ signal and NeuN staining 1 day after AQP4-IgG (top) or Ctrl-IgG (bottom) injection reveal persistence of neurons in the lesion area (scale bar = 40 µm). The yellow dashed line indicates the lesion border. Images are representative of similar experiments repeated independently in at least 3 animals.

To assess astrocytic behavior over time after selective astrocyte depletion, we used transgenic mice expressing the enhanced green fluorescent protein (eGFP) under the control of the pan-astroglial *Aldh1l1* promoter^19^. We have previously shown by acute ex vivo and in vivo two-photon imaging that human-derived recombinant AQP4-IgG efficiently binds and depletes mouse astrocytes within hours^20,21^. Similarly, stepwise intracortical microinjection of AQP4-IgG and complement in *Aldh1l1*^GFP^ mice (Fig. 1a) led to cylindrical lesions devoid of astrocytes (Fig. 1b) in the somatosensory cortex. Such lesions were absent after injection of complement with isotype control recombinant IgG (Ctrl-IgG; Fig. 1b). This selective astrocyte depletion did not affect the morphology and function of the vasculature as visualized after intravenous administration of Texas Red dextran (Fig. 1b,c). No overt changes in vessel density, average vessel diameter (Fig. 1d,e), resting capillary blood flow and capillary diameter measured by two-photon line scan imaging (Fig. 1f,g) were observed compared to Ctrl-IgG injections. Furthermore, immunohistological staining for the neuronal marker NeuN showed the presence of neuronal population within the astrocyte-depleted area (Extended Data Fig. 1).

**Extended Data Fig. 2:**
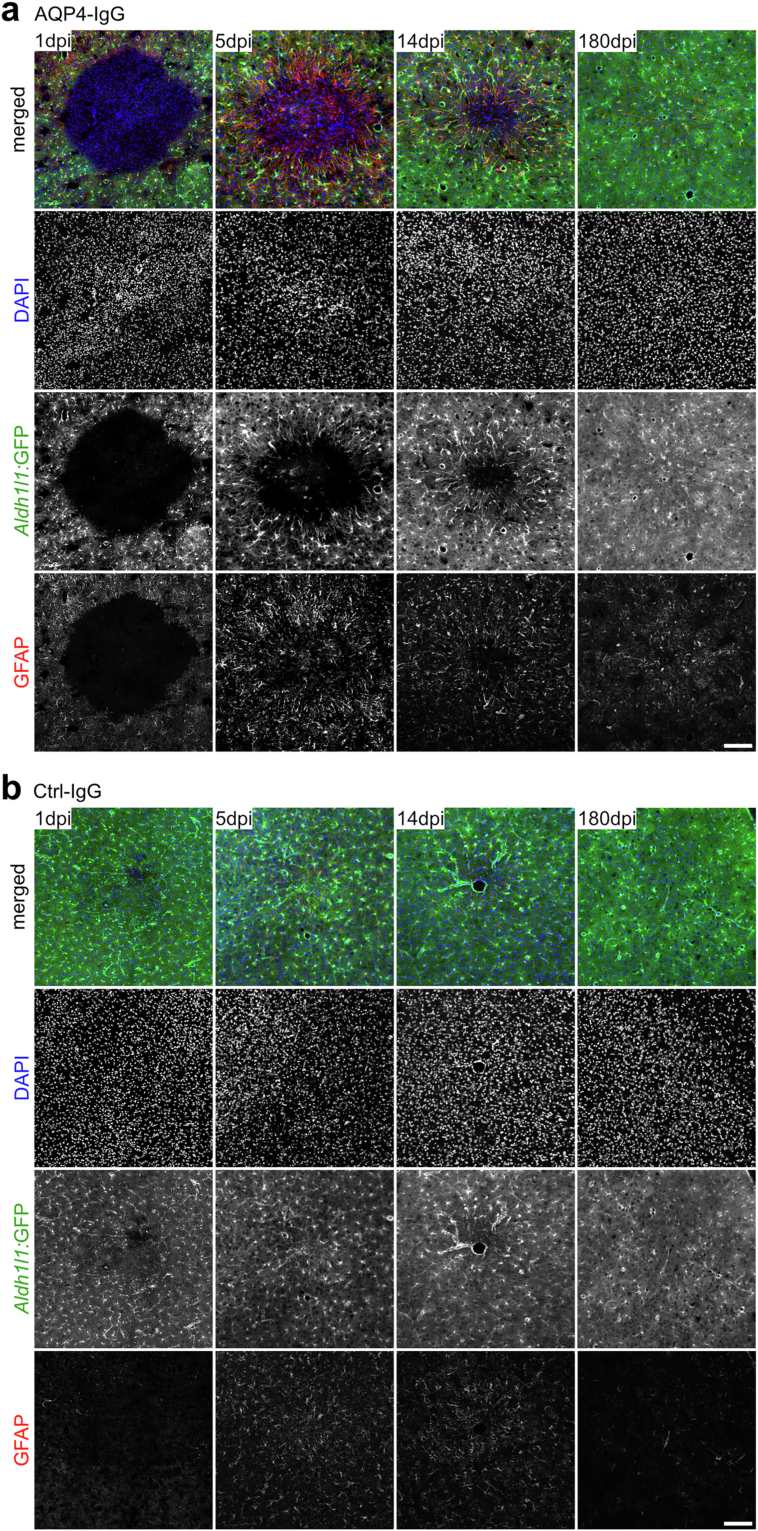
Immunohistochemical analysis of the AQP4-IgG-mediated lesion recovery over time. **a**, Composite confocal images and single channels of DAPI, astrocyte-endogenously expressed GFP, and GFAP in horizontal sections from representative AQP4-IgG-mediated lesions at different time points postinjection. **b**, The same as in **a**, from representative Ctrl-IgG injected areas. Scale bars = 100 µm. Images are representative of similar experiments repeated independently in at least 3 animals.

We then tracked astrocytic responses after local astrocyte depletion through longitudinal in vivo two-photon microscopy. We found that the area of astrocyte GFP signal loss was progressively restored within a few weeks (Fig. 1h,i). In parallel, the number of astrocytic cell bodies in the depleted area rapidly increased over time, reaching a plateau within a few weeks (Fig. 1j). Notably, focal ablation of astrocytes did not cause the formation of a glial border (Extended Data Fig. 2), as observed in animal models of traumatic brain injury or stroke^7,9^.

### Early structural remodeling of perilesional astrocytes

**Fig. 2:**
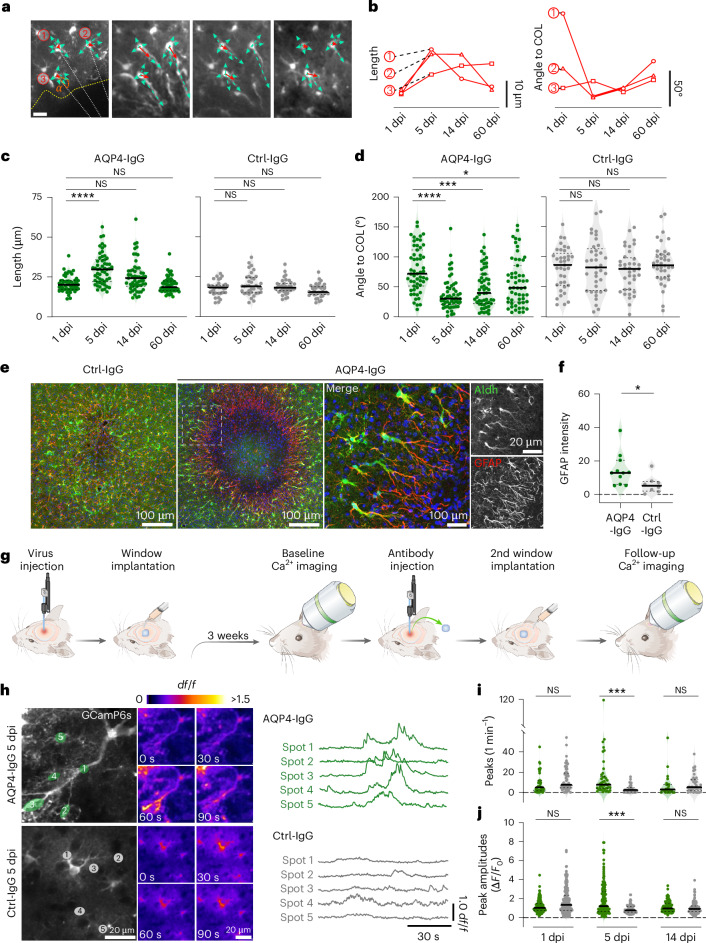
Astrocyte depletion-induced astrocyte reactivity shows early morphological and functional changes. **a**,**b**, Representative images (**a**) and traces (**b**) of three cells illustrating the vector-based analysis approach used for determination of the process length and angle *α* (orange); white dashed lines project to the COL and the yellow dashed line indicates the lesion border. Cyan arrows show individual vectors for main astrocytic processes and the respective mean vectors are indicated by red arrows. Scale bar = 20 µm (all panels in **a**). **c**,**d**, Vector-based quantification of astrocytic process lengths (**c**) and polarization (**d**) up to 60 dpi; *n* = 55 astrocytes from three to four lesions from three mice for AQP4-IgG, *n* = 37 astrocytes from four lesions from four mice for Ctrl-IgG. *P = 0.0428, ***P < 0.001, ****P < 0.0001; Friedman test followed by Dunn’s multiple comparisons test. **e**, Confocal images of immunohistochemical staining against GFAP of fixed brain tissue 5 days after AQP4-IgG or Ctrl-IgG injection. Right: higher magnification images from the perilesion of the boxed area in the overview image, illustrating the upregulation of GFAP (DAPI in blue). **f**, Corresponding quantification of GFAP area intensity (AQP4-IgG—*n* = 11 mice, Ctrl-IgG—*n* = 8 mice). *P = 0.0203; Mann–Whitney test, two sided. **g**, Schematic illustration of experimental workflow for imaging of astrocytic calcium activity in astrocytes before and after AQP4-IgG/Ctrl-IgG injection. **h**, Representative two-photon images of GCamP6s activity in astrocytes from an AQP4-IgG-injected and Ctrl-IgG-injected area 5 days after lesion induction (top and bottom, respectively). Color-coded images display calcium levels in the same field of view at different time points. Right: individual traces from the indicated spots (1–5). **i**,**j**, Quantification of calcium peak frequency (**i**) and maximum peak amplitudes (**j**) at 1, 5 and 14 dpi for AQP4-IgG compared to Ctrl-IgG (AQP4-IgG, 1 dpi—*n* = 39 cells from six mice, 5 dpi—*n* = 42 cells from six mice, 14 dpi—*n* = 27 cells from five mice; Ctrl-IgG, 1 dpi—*n* = 55 cells from seven mice, 5 dpi—*n* = 41 cells from six mice, 14 dpi—*n* = 34 cells from four animals. ***P < 0.001; Kruskal–Wallis test, two sided. Thick black lines represent the median, and fine black dashed lines represent the first and third quartiles. COL, center of the lesion. Schematic in **g** created in BioRender; Wyss, M. https://BioRender.com/n18jcee (2026). Source data

Astrocytes show a reactive phenotype characterized by heterogeneous morphological, transcriptional and functional changes on exposure to various pathological stimuli^4^. To assess the dynamics of the structural remodeling of surviving perilesional astrocytes in vivo, we performed a three-dimensional (3D) vector-based analysis of astrocyte process length and orientation of randomly chosen perilesional astrocytes (Fig. 2a,b). We found a robust elongation of astrocytic processes, reaching the maximum length at 5 days postinjection (dpi), followed by a recovery at 60 dpi (Fig. 2c). This was accompanied by the specific orientation of astrocytic processes toward the center of the lesion. In contrast to process elongation, this polarization remained stable within the 2-month observation period (Fig. 2d).

In line with these morphological changes, we found a transient upregulation of GFAP, a typical marker for astrogliosis^22^, in histological sections comparing AQP4-IgG and Ctrl-IgG areas (Fig. 2e,f), whose peak was reached within the first week, followed by a gradual normalization to control levels at later stages (Extended Data Fig. 2).

Astrocytes display distinct local calcium transients^23^ that have crucial roles in modulating neuronal activity, in promoting process outgrowth during development, and that may become dysregulated under pathological conditions^24^. To examine if calcium signaling might be altered in perilesional astrocytes during lesion repopulation, we recorded spontaneous astrocytic calcium transients, using an AAV-based GCaMP6s injected 3 weeks before AQP4-IgG/Ctrl-IgG injection (Fig. 2g). While at 1 dpi the spontaneous activity of perilesional astrocytes was similar in both conditions, at 5 dpi perilesional astrocytes developed a marked increase in calcium transient frequencies and amplitudes compared to astrocytes from Ctrl-IgG-injected areas (Fig. 2h–j and Supplementary Video 1). By 14 dpi, calcium dynamics had normalized, along with increased repopulation of the lesions (Fig. 2i,j). These results suggest that elevated calcium signaling may functionally reflect the extensive structural remodeling of perilesional astrocytes during lesion repopulation.

### Perilesional astrocytes undergo repeated cell divisions

**Fig. 3:**
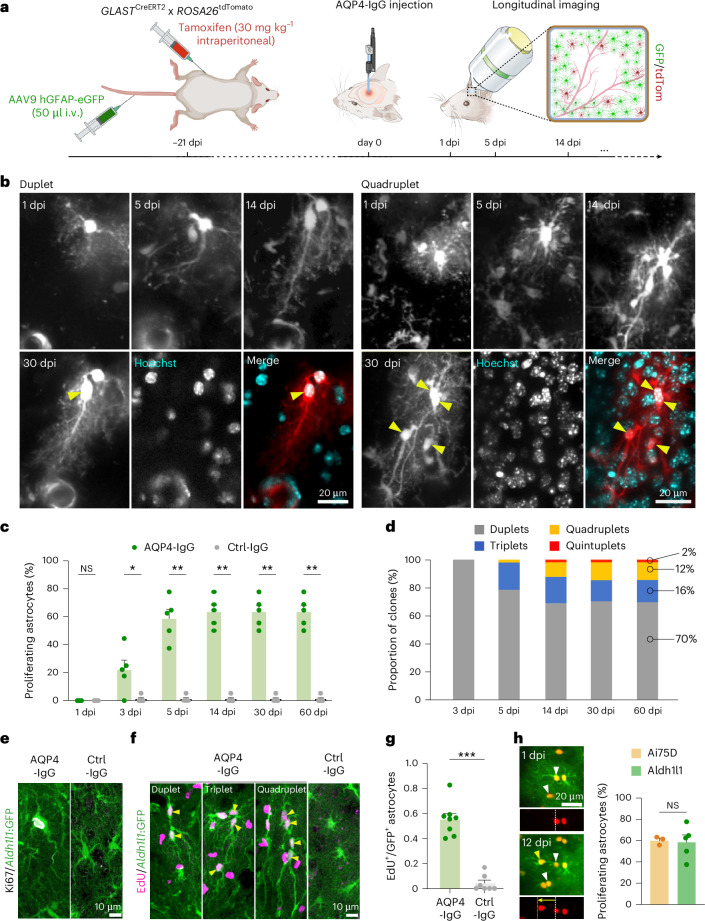
Perilesional astrocytes undergo multiple cell divisions. **a**, Sparse-cell labeling protocol for intravital two-photon imaging in *GLAST*^CreERT2^ x *ROSA26*^tdTomato^ mice allowing the monitoring of individual astrocytes over time. Schematic in **a** partly created in BioRender; Wyss, M. https://BioRender.com/ypewond (2026). **b**, Tracking single tdTom^+^ astrocytes over time allows detailed morphological evaluation and shows the proliferative activity of single perilesional astrocytes with one (left) or more (right) daughter cells (yellow arrowheads) counterstained in vivo at the last imaging time point (30 dpi) with the nuclear stain Hoechst 33342 (Supplementary Video 2). **c**, Percentage of proliferating perilesional astrocytes, *n* = 5 mice for both conditions, AQP4-IgG and Ctrl-IgG. *P = 0.0476, **P < 0.0079; Mann–Whitney test, two sided. **d**, Distribution of division cycles in proliferating perilesional astrocytes over time (*n* = 5 mice). **e**–**g**, Immunohistochemical staining for Ki67 (**e**) and EdU (**f**) in astrocytes from AQP4-IgG-injected and Ctrl-IgG-injected areas. Yellow arrowheads indicate EdU^+^ astrocytic clones. Ratio of EdU^+^ to all GFP^+^ perilesional astrocytes in both conditions (**g**); AQP4-IgG—*n* = 8 lesions from eight animals, Ctrl-IgG—*n* = 7 lesions from six mice. ***P < 0.001; Mann–Whitney test, two sided. **h**, Left: example from perilesional astrocytes 1 and 12 days after lesion induction in *GLAST*^CreERT2^ x *Ai75D* mouse line (Ai75D) illustrating proliferation of perilesional astrocytes (white arrowheads), and a translocation of a daughter nucleus (yellow arrowhead). Bottom: single channel showing the nuclear-localized tdTomato. Right: quantification of proliferating astrocytes in Ai75D mouse line compared to a cohort of *Aldh1l1*^GFP^ animals (*n* = 3 and 5 mice, respectively; Mann–Whitney test, two sided). Data are represented as mean ± s.e.m. Schematic in **a** partly created in BioRender; Wyss, M. https://BioRender.com/ypewond (2026). Source data

**Extended Data Fig. 3:**
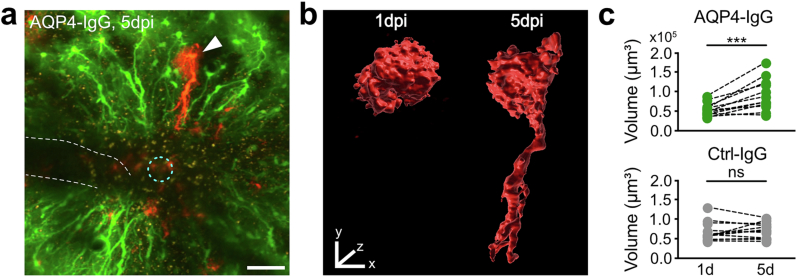
Territory change of single perilesional astrocytes. **a**, Sparse labeling of astrocytes in *GLAST*^CreERT2^ x *ROSA26*^tdTomato^ allows tracking of single tdTom^+^ perilesional astrocytes over time (white arrowhead) and detailed morphological evaluation, including astrocytic territory changes (cyan dashed circle: injection site; white dashed lines mark a vessel); scale bar: 50 µm. **b**, Three-dimensional surface rendering of a representative astrocyte (marked in **a**) at days 1 and 5 (scale bar: 20 µm in x, y, and z-dimensions). Images are representative of similar experiments repeated independently in 4 mice. **c**, Quantification of territory changes in AQP4-IgG versus Ctrl-IgG condition, n = 12 cells from 4 mice, respectively; ***p < 0.001, and ns: not significant (Wilcoxon signed-rank test, two-sided). Source data

Next, we wondered about the origin of newly appearing astrocytes in the lesions. Given that tracking single astrocytes with global *Aldh1l1*^GFP^ labeling is difficult, we sparsely labeled astrocytes with a low-dose tamoxifen injection in *GLAST*^CreERT2^ x *ROSA26*^tdTomato^ mice in combination with an intravenous injection of PHP.eB/2-hGFAP-EGFP viruses to achieve widespread astrocytic labeling to precisely localize the lesion borders (Fig. 3a). Five days after lesion induction, the territory occupied by a single astrocyte increased by ~70%, while the astrocyte domains from control areas remained stable (Extended Data Fig. 3a–c), corroborating the observations made in *Aldh1l1*^GFP^ mice.

By tracking individual perilesional astrocytes over several weeks, we observed focal swellings within the thick processes at varying distances from the astrocytic cell bodies (Fig. 3b), which were suggestive of nuclear structures. Topical application of the nuclear dye Hoechst 33342 at 30 dpi confirmed that these structures contained nuclei (Fig. 3b and Supplementary Video 2), revealing a substantial proliferative activity in perilesional astrocytes.

With this longitudinal analysis, we could trace the newly appearing intralesional astrocytes back to their corresponding perilesional mother cells and found that approximately 60% of all perilesional astrocytes proliferated within 60 days after the lesion induction (Fig. 3c), compared to only 1% within Ctrl-IgG areas. This proliferation was not restricted to the juxtavascular niche (defined as astrocytic soma <5 µm away from a vessel wall) and could undergo several rounds of mitosis, generating up to four daughter cells (Fig. 3d).

Immunohistochemical staining with the proliferation marker Ki67 confirmed astrocytic proliferation (Fig. 3e) in AQP4-IgG compared to Ctrl-IgG areas. However, Ki67 only detects cells that are in active phases of proliferation. To assess the proliferative capacity over a longer period of the repopulation phase, we treated mice with the nucleoside thymidine analog 5-ethynyl-2’-deoxyuridine (EdU) for 7 days after lesion induction. The EdU detection using co-immunolabeling of endogenous *Aldh1l1*^GFP^ and GFAP in fixed tissue revealed EdU^+^ perilesional astrocytes in AQP4-IgG areas (Fig. 3f,g). Again, a variable number of daughter cells could be observed in perilesional areas. In line with the in vivo data, around 50–60% perilesional astrocytes were EdU^+^ at 14 dpi (Fig. 3g). To directly track nuclear division, we used the *GLAST*^CreERT2^ x *Ai75D* mouse line, which expresses nuclear-localized tdTomato upon tamoxifen induction, confirming their proliferative activity in vivo (Fig. 3h). These results demonstrate a substantial proliferative capacity of cortical astrocytes in the adult brain under conditions of selective and focal astrocyte loss, which is not restricted to the vascular niche and not limited to a single-cell division.

### Perilesional multinucleated astrocytes undergo nucleokinesis during lesion repopulation

**Fig. 4:**
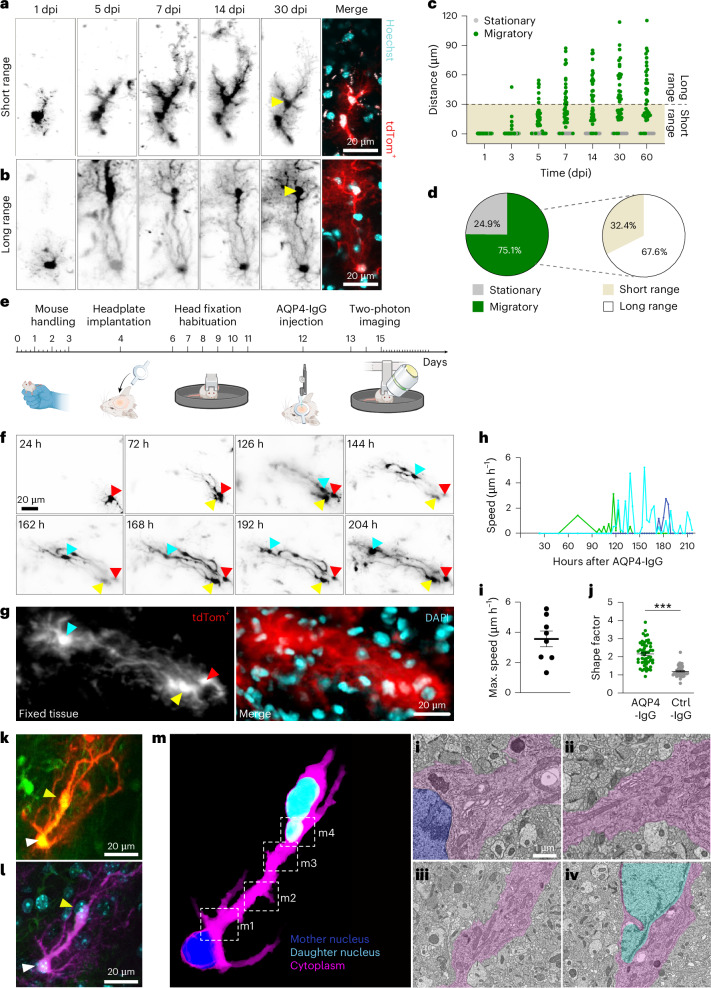
Multinucleated astrocytes use nucleokinesis for lesion repopulation. **a**,**b**, Examples of tdTom^+^ astrocytes undergoing mitosis followed by short-range (**a**) or long-range (**b**) translocation of daughter-cell nuclei (yellow arrowheads). Nuclear counterstaining is shown at last imaging time point (30 dpi) with Hoechst 33342 in vivo. **c**, Distance of adult-born astrocyte nuclei from their mother cells over time (*n* = 61 cells from six mice). Dashed line indicates the threshold for short-range and long-range displacement. **d**, Frequencies of different behaviors of proliferating astrocytes (data from **c**). **e**–**j**, High-frequency time-lapse imaging in awake mice revealing saltatory movement of adult-born astrocytic nuclei. Experimental workflow (**e**). **f**, Time lapses of an individual perilesional astrocyte (red arrowhead; Supplementary Video 3), showing its remodeling and proliferation, giving birth to two progenies (cyan and yellow arrowheads). The yellow arrowhead indicates the stationary daughter cell and the cyan arrowhead shows the migratory daughter nucleus. **g**, The astrocyte from **f** and its daughter cell are identified in postfixed tissue and counterstained with DAPI. Examples of speed traces of individually tracked astrocytic nuclei (**h**) as well as distribution of speed maxima (**i**; *n* = 7 nuclei from four mice; thick black line represents mean, and thin black lines represent s.e.m.). **h**, Cyan-colored lines indicate the nuclei shown in cyan in **f**. The elongated shape of the nucleus during nucleokinesis (**f**) is noted. **j**, Change in nuclear shape in astrocytes 5 dpi in AQP4-IgG-IgG (*n* = 52 nuclei from five mice) and Ctrl-IgG-injected areas (*n* = 48 from four mice) in fixed tissue. ***P < 0.001; Mann–Whitney test, two sided. Data are represented as mean ± s.e.m. **k**, In vivo two-photon *z*-projected stack of a repopulating astrocyte (white arrowhead) with translocated daughter nucleus (yellow arrowhead) 10 days after AQP4-IgG injection. **l**, Two-photon image of the cell pair from **k** in postfixed tissue, counterstained with DAPI (cyan). Scale bars = 20 µm. **m**, The 3D surface rendering of the cell pair from **k** and **l** obtained by volume correlative light and electron microscopy with array tomography analysis. i–iv, representative EM sections (boxed areas in **m**, demonstrating that mother and daughter nuclei share the same cytoplasm). The EM segmentation is representative of at least three biological replicates. Schematic in **e** created in BioRender; Wyss, M. https://BioRender.com/fmu6gzg (2026). Source data

We then wanted to visualize astrocyte repopulation dynamics with high temporal resolution in vivo. Repetitive imaging of individual mature proliferating astrocytes revealed daughter-cell nuclei at large distances from their mother cells (Fig. 4a–c). The radius of cortical astrocytic domains typically ranges between 20 and 30 µm^25^. Based on these previously defined astrocytic domains, we grouped daughter cells into two categories—those with a short-range displacement (<30 µm) and those with a long-range displacement (>30 µm). Translocation activity was highest during the first 14 days after AQP4-IgG injection (Fig. 4c). Approximately 75% daughter-cell nuclei relocalized within the first 7 days, of which >60% (±4.7) moved more than 30 µm away from their mother cell (Fig. 4d). In all Ctrl-IgG areas, only one single-cell nucleus relocalized.

Daily two-photon imaging sessions were insufficient to precisely track the movement patterns and speeds of translocating daughter-cell nuclei. To overcome this limitation, we applied a high-frequency two-photon imaging protocol in awake mice (Fig. 4e). We imaged the mice every 3 h over six consecutive days, precisely capturing the dynamics of daughter nuclei (Fig. 4f–h). Interestingly, adult-born daughter-cell nuclei were squeezed through process extensions of mother cells in a saltatory manner (Fig. 4f–h and Supplementary Video 3). The maximum speed of this process reached 3.5 µm h^−1^ (Fig. 4i) with peak rates of 5.6 µm h^−1^ and complete stops in between for up to 9 h. The moving nuclei adopted an ellipsoid shape, regaining their circular form on completion of translocation (Fig. 4f). Analysis of the nuclear shape by DAPI staining in fixed tissue at the time of maximum movement activity (5–8 dpi) confirmed that the nuclei of intralesional astrocytes had a higher shape factor (length-to-width ratio) compared to control regions (Fig. 4j).

During development, nucleokinesis is a critical part of newborn NPC migration, where the orchestrated extension of leading processes and movement of cell bodies and nuclei enable guided migration to populate the neocortex^26^. After separation from the mother cell, NPCs use long radial glial fibers as guidance to find their final location^27^. We thus wondered whether the observed nucleokinesis in perilesional astrocytes shows similar mechanisms. Given the resolution limits of light microscopy, we used correlative light and electron microscopy followed by serial-section electron microscopy to evaluate the ultrastructure of repopulating astrocytes. EM segmentation of perilesional astrocytes with long-range daughter-cell translocation and careful tracking of their membranes at 10 dpi revealed that, despite their distance from each other, mother and daughter nuclei share the same cytoplasm (Fig. 4k–m). Daughter-cell nuclei again had an elongated shape (*n* = 3 mice) and sometimes nuclear blebbing (Fig. 4m), as is known to transiently occur during nucleokinesis in multiple cell types^28,29^. These findings suggest against NPC-like migration, showing instead that perilesional astrocytes, following mitosis, remain in a multinucleated intermediate phase, squeezing and guiding daughter-cell nuclei to new astrocytic territories.

**Extended Data Fig. 4:**
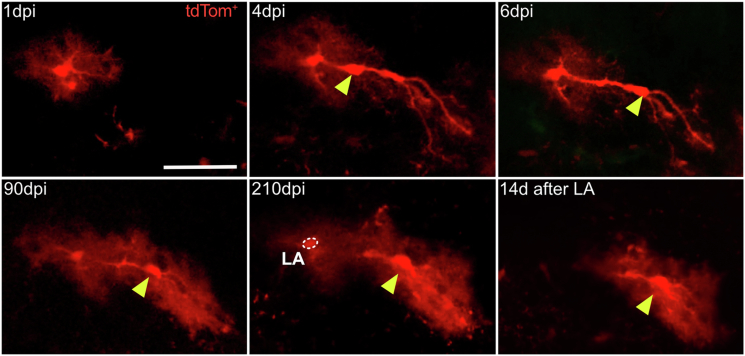
Daughter cell viability is uncoupled from mother cell fate. In vivo time-lapse imaging of a single tdTom^+^ perilesional astrocyte giving rise to one progeny (yellow arrowhead), which migrates through nucleokinesis to establish itself in a new territory. Over time, the daughter cell develops increased branch complexity. The dashed circle indicates the site of laser ablation (LA) targeting the mother cell. Bottom right, confirmation of the successful ablation of the mother cell and the survival of the daughter cell 2 weeks after LA (scale bar = 50 µm).

To determine whether cytokinesis ultimately occurs, we selectively ablated mother cells using laser ablation after monitoring mother–daughter pairs for several months. Six of seven daughter cells (85.7%) survived after the ablation of their mother cells, indicating cellular independence at later stages (Extended Data Fig. 4). However, this approach does not provide precise information on the timing of cytokinesis.

Together, these observations describe a mode of astrocyte repopulation involving prolonged multinucleated states and the gradual translocation of daughter nuclei through shared cytoplasmic structures to occupy previously depleted astrocyte territories.

### Perilesional astrocytes display a transient injury-associated gene signature

**Fig. 5:**
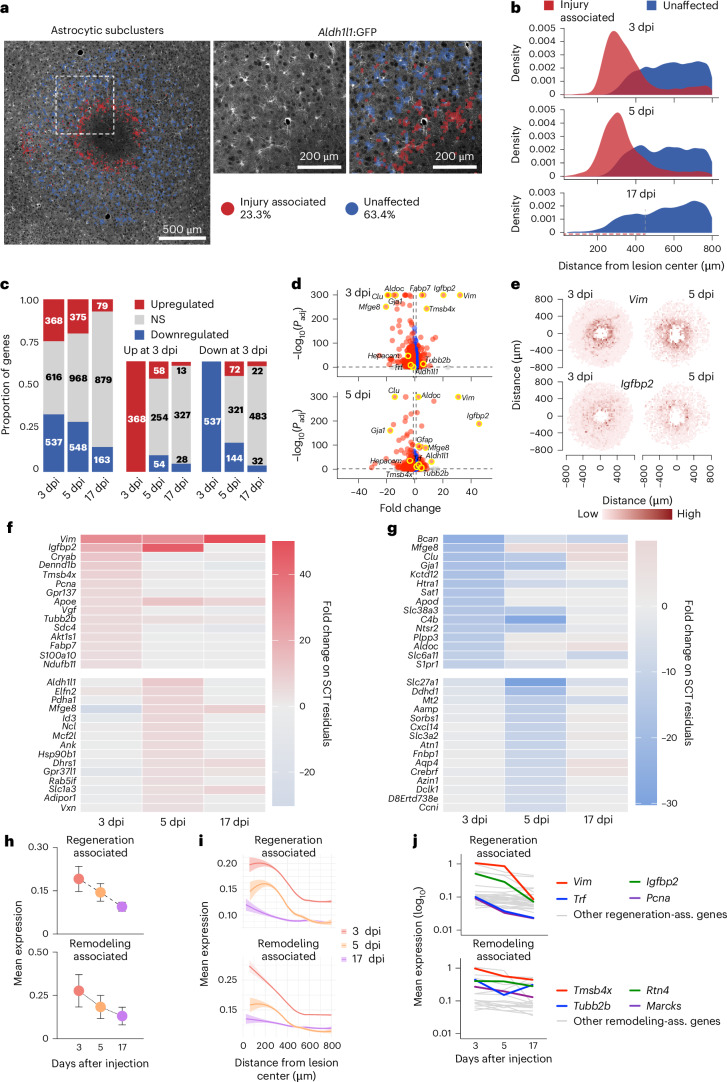
Spatiotemporal transcriptomic analysis reveals transient injury-associated signature. **a**, Spatial distribution of astrocytic subclusters at 3 dpi. Left: overlay of ST spots on a confocal image of endogenously expressed astrocytic GFP (*Aldh1l1*^GFP^ mouse line). Middle and right: zoom-in of the boxed area above with single GFP channel (middle) and with respective overlay of astrocytic clusters (right). Image is representative of similar results observed in seven lesions from four mice. **b**, Spatial density distribution of injury-associated versus unaffected astrocyte clusters over time. Bottom: the mean distance of 75th percentile of injury-associated cluster distribution at earlier time points is indicated by red dashed line. **c**, Number of significantly upregulated, downregulated or not regulated genes (of 1,845) at each time point after lesion induction (left). Right: temporal evolution of DEGs initially identified as upregulated or downregulated at 3 dpi. **d**, Volcano plot comparing injury-associated with unaffected astrocytes at 3 dpi. Vertical position reflects significance (−log_10_ (*P*_adj_)). Red dots indicate significantly upregulated or downregulated genes. Wilcoxon rank-sum test with BH correction for multiple comparisons. **e**, Spatial projection of selected top upregulated genes on the coordinate system with 0 representing the lesion core (3 and 5 dpi). Color gradient indicates the mean gene expression level. **f**, Heatmap showing fold change of the top 15 upregulated genes. **g**, Downregulated genes in the injury-associated astrocyte cluster at 3 and 5 dpi across time. **h**, Dynamics of the mean expression of regeneration-associated and remodeling-associated DEGs across time. **i**, Density plot of mean expression levels for regeneration-associated (top) and remodeling-associated DEGs (bottom) depending on distance from the lesion center at 3, 5 and 17 dpi (3 dpi—*n* = 7 lesions from four mice; 5 dpi—*n* = 4 lesions from two mice; 17 dpi—4 lesions from two mice). Data are represented as mean ± s.e.m. **j**, Mean expression levels of individual genes in the injury-associated astrocyte cluster over time. Selected genes are color-coded, the remaining regeneration- and remodeling-associated genes are shown in gray. BH, Benjamini–Hochberg; ass., associated. Source data

**Extended Data Fig. 5:**
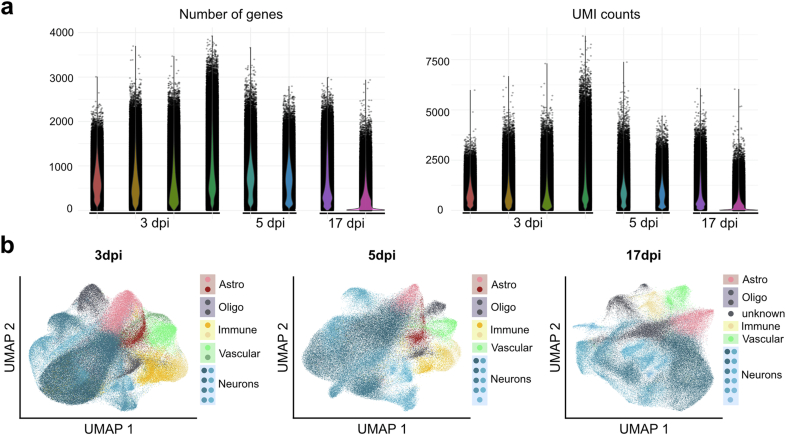
Spatial transcriptomics of the cortical tissue containing astrocyte-depleted lesions. **a**, Violin plots showing the number of detected genes (left) and UMI counts (right) for each sample at the given time points. **b**, Unsupervised UMAP visualization of identified clusters at different time points, colored by cell type, astro = astrocytes, oligo = oligodendrocytes, immune = immune cells, vascular = vascular cells.

Next, we considered how the perilesional astrocytes observed in our AQP4-IgG-mediated model change their transcriptional programs, which accompany their cytoskeletal remodeling and nuclear translocation. To investigate the temporal transcriptional dynamics of perilesional astrocytes, we performed spatial transcriptomic (ST) profiling using the Visium HD 10x platform. Axial sections of cortical layer 2/3 encompassing lesion sites were obtained from *Aldh1ll*^GFP^ mice at 3, 5 and 17 days after lesion induction. The sequencing yielded a dataset with an average of 1,648,804,428.13 reads per sample, detecting an average of 16,600.75 genes per sample. After quality control (Methods; Extended Data Fig. 5a), an area of 800 µm radius surrounding the lesion center was further analyzed to capture spatially resolved gene expression patterns (Fig. 5a). Unsupervised clustering based on the top 20 principal components identified 16 distinct clusters, belonging to five distinct cell types at 3 dpi and 5 dpi, and 17 clusters at 17 dpi (Extended Data Fig. 5b).

**Extended Data Fig. 6:**
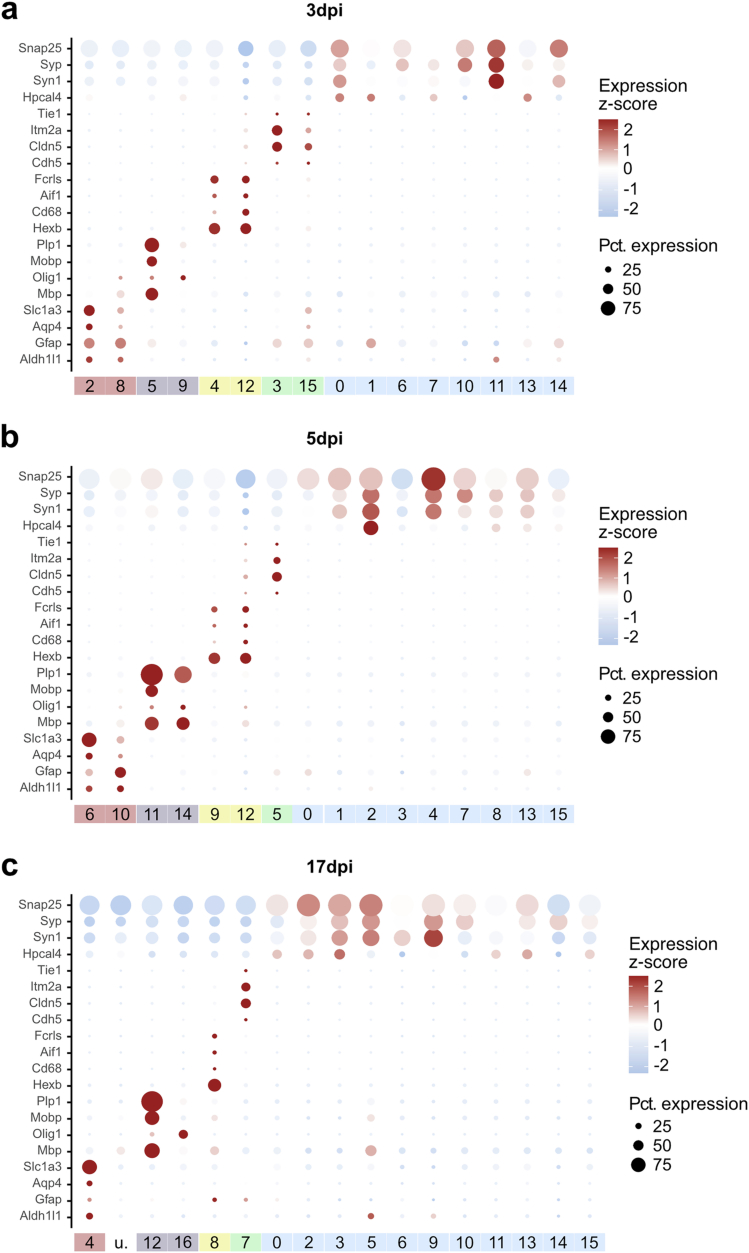
Cell cluster identification by expression of canonical cell-type markers. **a**–**c**, Dot plots, for 3 dpi (**a**), 5 dpi (**b**), and 17 dpi (**c**) time points, showing expression of cell-type-specific markers (astrocyte clusters: 2 and 8 at 3 dpi, 6 and 10 at 5 dpi, and cluster 4 at 17 dpi). Color gradient indicates normalized gene expression level. u. = unknown. Source data

By focusing on clusters expressing canonical markers such as *Aldh1l1, Gfap, Aqp4, Gja1* and *S100b*, we identified two distinct astrocyte clusters (clusters 2 and 8 at 3 dpi, clusters 6 and 10 at 5 dpi; Extended Data Fig. 6a–c) and one astrocyte cluster at 17 dpi (cluster 4). These were further filtered and validated by spatially mapping the clusters onto confocal images of tissue with pan-astrocytic GFP expression.

Spatial integration revealed that cluster 8 at 3 dpi is localized specifically at the lesion border, while cluster 2 represents astrocytes located further away from the lesion core (Fig. 5a,b). Comparing the RNA profiles of these clusters with previously published reference astrocyte gene panels^30–35^ revealed enrichment of healthy astrocyte-associated genes in cluster 2 at 3 dpi and 6 at 5 dpi. In contrast, genes associated with injury-associated astrocytes were predominantly enriched in cluster 8 at 3 dpi and cluster 10 at 5 dpi. Spatial mapping further supported these findings, showing that the injury-associated cluster was primarily confined to the lesion rim (Fig. 5a,b). To compare astrocyte transcriptomes in the perilesional area over time, we included 17-dpi astrocytes located between the lesion center and 425.5 µm into the analysis (that is, the mean of the 75th percentile from the density plots of clusters 8 and 10 at 3 and 5 dpi in Fig. 5b). Strikingly, concomitant with the completion of astrocyte repopulation (17 dpi), the injury-associated genetic profile was no longer detectable (Fig. 5b).

For a deeper characterization of the molecular changes in perilesional astrocytes, we analyzed differentially expressed genes (DEGs) between injury-associated and unaffected astrocyte clusters. At day 3, 1,551 identified DEGs (59.6%) were significantly upregulated or downregulated in injury-associated astrocytes; a similar proportion (48.7% of 1,899) was observed at day 5, while only 21.5% genes remained differentially expressed by day 17 (of 1,123 DEGs and only 7.0% upregulated), indicating a transient activation state (Fig. 5c). Among the top DEGs enriched in injury-associated astrocytes across time were classical reactivity markers such as *Vim* and *Gfap*, and gliogenic genes such as *Trf*, *Igfbp2*, *Pcna and Mfge8* (Fig. 5d,e). Notably, *Mfge8* displayed a distinct biphasic expression pattern, characterized by an initial downregulation, followed by a rapid upregulation at the peak of astrocyte activation. Within the astrocyte lineage, *Mfge8* serves diverse functions—it not only promotes progenitor proliferation and migration during development, but also acts as a gatekeeper of progenitor activation in the subventricular zone, orchestrates debris clearance in the crosstalk with microglia and contributes to the reactive phenotype in normal aging^36,37^. This dynamic regulation of *Mfge8* reflects its context-dependent function in modulating astrocyte activation.

**Extended Data Fig. 7:**
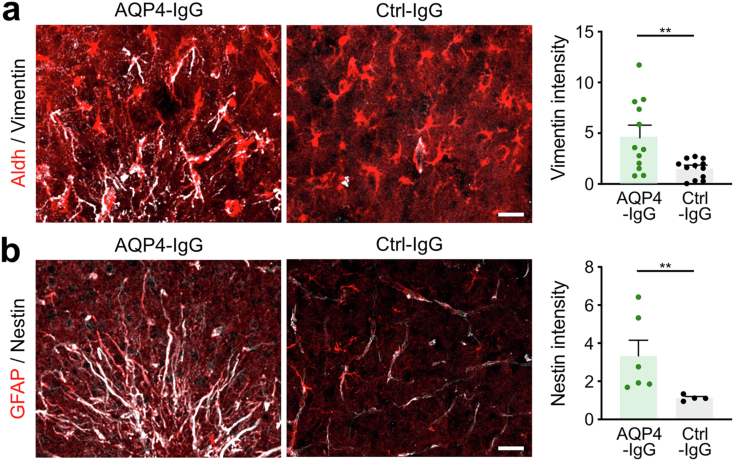
Repopulating astrocytes express developmental markers. **a**,**b**, Left and middle, representative confocal images of fixed AQP4-/Ctrl-IgG-injected areas from *Aldh1l1*^GFP^ mice 14 dpi, stained with the developmental markers vimentin in **a** and nestin in **b** (in white, scale bar = 20 µm). Please note the nestin-positive vasculature in **b**. Right, corresponding quantification of vimentin and nestin area intensity (AQP4-IgG: n = 12 lesions from 12 animals, Ctrl-IgG: n = 12 lesions from 11 animals for vimentin; AQP4-IgG: n = 6 lesions from 5 animals, Ctrl-IgG: n = lesions from 4 animals for nestin). Vimentin: **p = 0.0083; nestin: **p = 0.0095, Mann–Whitney test, two-sided. Data are represented as mean ± s.e.m. Source data

Moreover, *Tmsb4x* and *Tubb2b* were also significantly upregulated (Fig. 5f). These genes are involved in active cytoskeletal remodeling and are known to promote neural progenitor migration during development^38,39^ and tumor cell migration in several cancers, including glioma^40^. This indicates that repopulating astrocytes activate actin-associated and microtubule-associated structural remodeling programs, including nucleokinesis, during lesion recovery. Prominently downregulated genes included canonical astrocyte markers, such as *Aldoc*, *Aqp4* and *Clu;* gap junction-associated gene *Gja1*; branch complexity-associated gene *Hepacam*; and further genes related to synapse regulation and metabolism, such as *Plpp3* (Fig. 5d,g). Immunohistochemical analysis confirmed an upregulation of some developmental proteins^10^, such as GFAP (Fig. 2e–f), vimentin and nestin, in repopulating astrocytes (Extended Data Fig. 7).

**Extended Data Fig. 8:**
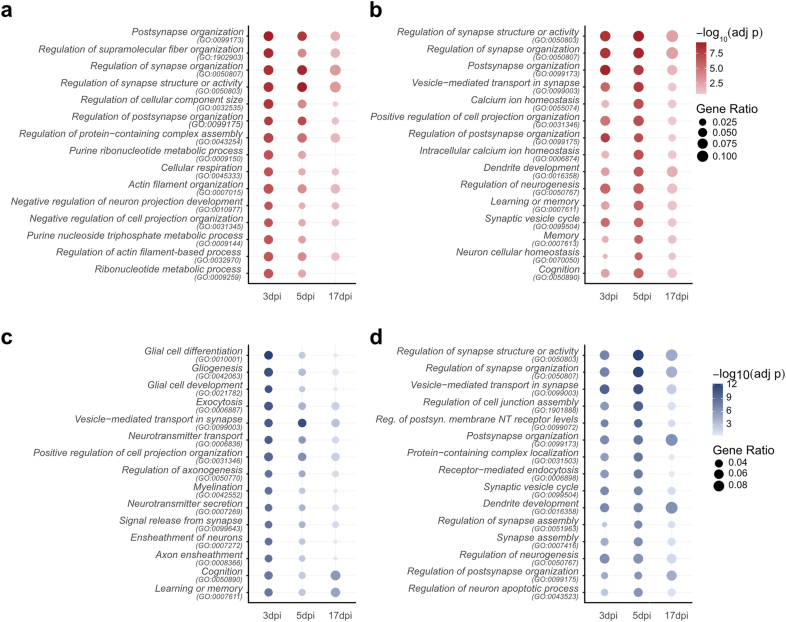
Astrocyte depletion-associated upregulated and downregulated pathways. **a**,**b**, Dot plots showing top 15 upregulated GO terms (biological process, based on p-adjusted values) in the injury-associated cluster at 3 dpi (**a**) and 5 dpi (**b**). **c**,**d**, Top 15 downregulated GO terms (biological process, based on p-adjusted values) at 3 dpi (**c**) and 5 dpi (**d**). Dot size indicates gene ratio, color represents adjusted p-value. Source data

Gene Ontology (GO) analysis revealed significant enrichment of terms related to structural remodeling of cellular size and cytoskeletal architecture, and to the regulation of gliogenesis/neurogenesis within the injury-associated astrocyte cluster (Extended Data Fig. 8), suggesting a role for astrocyte remodeling during lesion recovery. Consistent with the identity of gray matter astrocytes, terms associated with synapse function regulation were also highly enriched.

To further characterize the transcriptional signature of perilesional astrocytes during lesion recovery, we analyzed a set of DEGs functionally associated with gliogenesis, regenerative activity and remodeling using a cassette of genes derived from gene set enrichment analysis, GO analysis pathway and literature sources (Supplementary Table 1). This revealed a time-dependent transcriptional trajectory, characterized by the early upregulation of plasticity-associated genes such as *Vim*, *Igfbp2*, *Trf* and *Grn*, followed by a marked downregulation of *Bcan*, *Htra1* and *Luzp2* (Fig. 5h–j and Source Data Fig. 5), implicating a tightly regulated, transient astrocyte activation program. Notably, *Htra1* acts as a counter-regulator of *Igfbp2*, encoding a serine protease that cleaves *Igfbp2* and thereby has a pivotal role in fine-tuning IGF signaling^41,42^.

The spatiotemporal integration of the remodeling-associated panel revealed that astrocytes showed a pronounced early upregulation of various plasticity-associated DEGs. Notably, upregulated genes included *Vim*, *Tmsb4x* and *Marcks*, which are involved in cytoskeletal remodeling, as well as *Tubb2b*, *Rtn4 and Tuba1a*, which are, among other functions, associated with nucleokinesis during development (Fig. 5h–j and Supplementary Table 1). In line, genes such as *Hepacam*, which is implicated in astrocytic branch complexity, were significantly downregulated (Fig. 5d). This distinct transcriptional activation program peaked at day 5 and was most pronounced around ~250 µm from the lesion center—corresponding to the region where surviving perilesional astrocytes reside (Fig. 5i). By day 17, expression of most nucleokinesis-related genes had returned to baseline levels (Fig. 5h–j). These transcriptome dynamics support a tightly regulated, transient activation of a remodeling program after focal astrocyte loss, which diminishes as astrocyte repopulation of the lesion site progresses.

### Connexin signaling is not required for astrocyte repopulation

**Extended Data Fig. 9:**
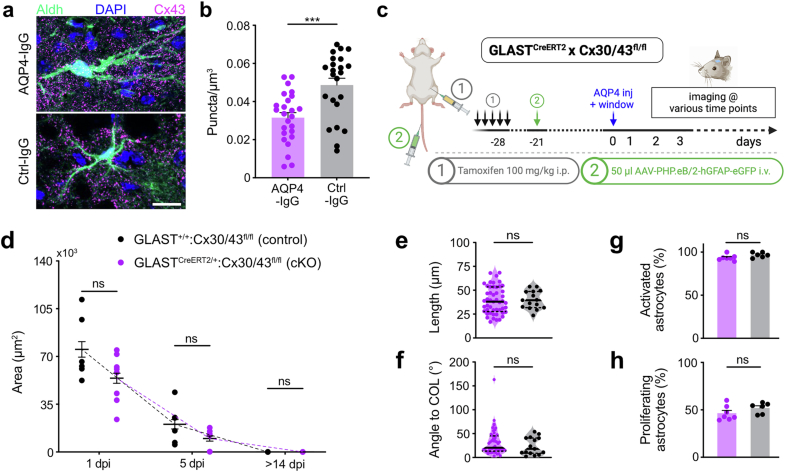
Repopulation of astrocyte-depleted areas is independent of gap junction coupling. **a**, Confocal images of representative astrocytes in AQP4-/Ctrl-IgG-injected areas from postfixed *Aldh1l1*^GFP^ mice, stained with Cx43 (scale bar = 10 µm). **b**, Ratio of Cx43 puncta per astrocyte domain from fixed tissue (AQP4-IgG: n = 25 cells from 6 animals, Ctrl-IgG: n = 23 cells from 6 animals; Mann–Whitney test, two-sided). Data represent mean ± s.e.m. **c**, Experimental approach to study the contribution of astrocyte-specific Cx30 and Cx43 on astrocytic repopulation. Schematic in **c** created in BioRender; Wyss, M. https://BioRender.com/ck8jtwd (2026). **d**, Time course of lesion size from connexin knockout mice (cKO) versus littermate controls in vivo after AQP4-IgG injection. n = 8 lesions from 6 cKO mice; n = 6 lesions from 4 control mice. ns = not significant (Mann–Whitney test, two-sided). Data represent mean ± s.e.m. **e**,**f**, Vector-based quantification of process length and polarization of perilesional astrocytes in cKO versus littermate controls (cKO: n = 49 astrocytes from 7 animals, controls: n = 15 astrocytes from 3 animals; Mann–Whitney test, two-sided). Thick black line represents the median, and fine black dashed lines represent the first and third quartiles. **g**,**h**, Percentage of activated and proliferating astrocytes is comparable between cKO and control mice (cKO: n = 7 lesions from 6 animals, controls: 6 lesions from 4 animals; Mann–Whitney test, two-sided). Data represent mean ± s.e.m. ***p < 0.001, ns = not significant. Source data

During development, glial progenitor cells are known to express different connexins, especially Cx43^43^, which is not only crucial for gap-junctional communication but also supports neuronal migration by providing connexin adhesive contacts^44^. In the adult brain, mature astrocytes express Cx30 and Cx43 subforms of gap junctions, allowing direct exchange of neurotransmitters and metabolites for intercellular communication^45^. However, our transcriptomic analysis indicated reduced expression of gap junction-associated genes. In line with this, the immunohistochemical analysis of Cx43 expression in perilesional astrocytes in AQP4-IgG areas compared to astrocytes in Ctrl-IgG areas from *Aldh1l1*^GFP^ mice showed a decrease in the density of Cx43 expression (Extended Data Fig. 9a,b). To further assess whether connexin signaling contributes to astrocyte repopulation, we used recently generated inducible astrocyte-specific double Cx30 and Cx43 conditional knockout mice^46^, in which connexins are completely depleted from astrocytes (Extended Data Fig. 9c). Connexin knockout mice revealed no difference in lesion size, recovery (Extended Data Fig. 9d) and no difference in percentage of process elongation or polarization (Extended Data Fig. 9e,f). In addition, the number of activated astrocytes and the proliferation rate remained comparable to littermate controls (Extended Data Fig. 9g,h). These findings suggest that connexin signaling is not required for the observed astrocyte remodeling and lesion repopulation after focal astrocyte loss.

### Astrocyte lesion repopulation is limited in border-forming lesions

**Fig. 6:**
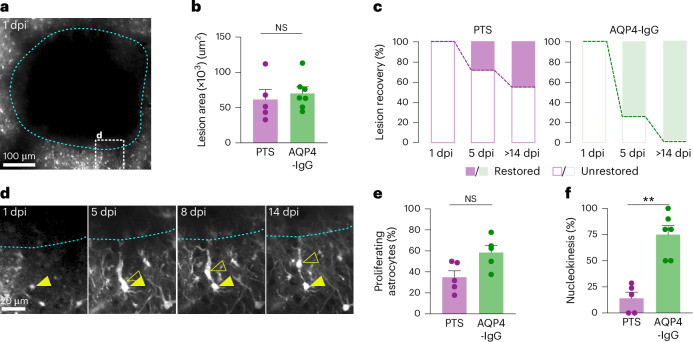
Astrocyte repopulation is limited in stroke lesions. **a**, Representative in vivo two-photon overview image of a PTS lesion at 1 dpi with astrocyte loss (cyan dashed area) in *Aldh1l1*^GFP^ mice. Image is representative of similar results observed in five lesions from five mice. White dashed box magnified in **d**. **b**, Quantification of lesion size. **c**, Percent of astrocyte lesion recovery over time in stroke lesions compared to AQP4-IgG-mediated lesions (PTS—*n* = 5 animals, AQP4-IgG–*n* = 7 lesions from six animals; Mann–Whitney test, two sided). **d**, Time lapses of magnified area from **a**, illustrating an individual perilesional astrocyte (filled yellow arrowheads) that proliferates, giving birth to a progeny whose nucleus is translocated for a short-range distance toward the lesion core (unfilled yellow arrowheads). The cyan dashed line indicates the lesion border. **e**, The percentage of proliferating (*n* = 5 animals for both conditions). **f**, Repopulating perilesional astrocytes in stroke lesions relative to AQP4-IgG-mediated lesions (PTS—*n* = 5 animals, AQP4-IgG—*n* = 6 animals; Mann–Whitney test, two sided). Data are represented as mean ± s.e.m. **P < 0.0022. PTS, photothrombotic stroke. Source data

Having characterized astrocyte lesion repair dynamics in astrocytopathy-driven neuroinflammation, we wondered whether similar remodeling and repopulation dynamics occur in border-forming astrocytes that surround the necrotic lesion core after CNS traumatic injury or stroke^7,32^. For better comparability with our selective astrocyte ablation model, we used a Rose Bengal photothrombosis protocol^47,48^ that produced focal cortical stroke lesions of similar dimensions to those induced by AQP4-IgG-mediated ablation (Fig. 6a,b). As expected for stroke lesions with glial border formation, astrocytic repopulation of the lesion areas remained incomplete (Fig. 6c) despite similar proliferative activity compared to AQP4-IgG-mediated lesions (Fig. 6d,e). Indeed, tracking individual proliferating astrocytes at the lesion border up to 14 days after stroke, a time point by which border formation is largely complete^7,32^, we detected nucleokinesis of daughter-cell nuclei only in a small fraction of cases (17.6% of all proliferating astrocytes; Fig. 6d,f) and these were all restricted to short-range translocations (range, min–max = 12.7–30.0 µm versus AQP4-IgG = 10.5–113.5 µm). These findings indicate that, despite comparable proliferative activity, astrocyte nuclear translocation and lesion repopulation are markedly reduced in border-forming CNS lesions.

### Ablation volume influences astrocyte remodeling responses

To test whether lesion volume influences astrocyte remodeling and repopulation responses, we chose an alternative approach that fulfills two requirements: (1) it causes selective astrocyte ablation in an area of similar size in the same brain region, and (2) it is not associated with the induction of a hypoxic environment and/or glial border formation.

**Fig. 7:**
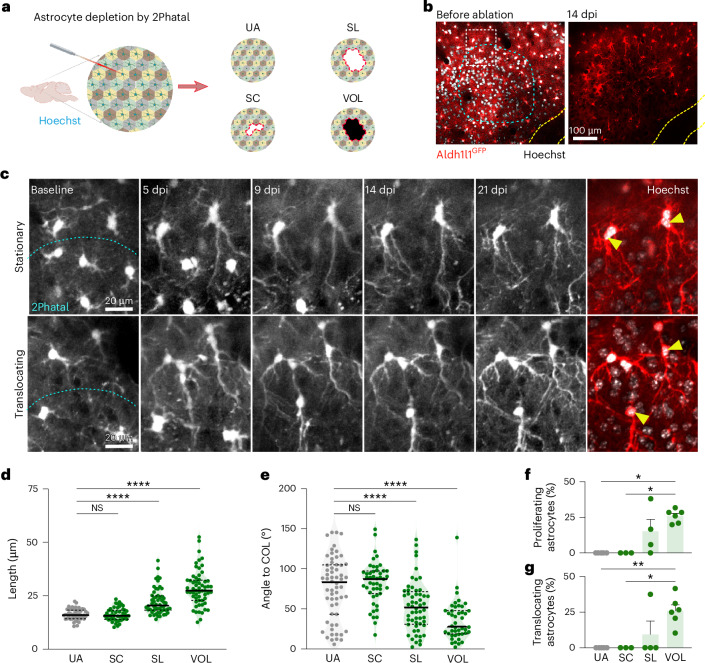
Astrocyte nucleokinesis is inducible by volume-targeted 2Phatal approach. **a**, Experimental setup for astrocyte ablation by modified 2Phatal method in different configurations—UA, that is, area without ablation but with in vivo Hoechst staining, SC (~5 cells, 0.08–0.14 × 10^6^ µm^3^), SL (~1.3–2.2 × 10^6^ µm^3^), VOL (~6.3–10 × 10^6^ µm^3^). **b**, Overview of the ablation region in *Aldh1l1*^GFP^ mice before (left) and 14 days after 2Phatal laser ablation (right). The cyan dashed line encompasses the ablation area, white dashed square indicates region of zoom-ins shown in **c**. In vivo Hoechst-33342-stained nuclei for 2Phatal induction are shown in white; astrocytes are shown in red, yellow dashed lines indicate a nearby blood vessel. Image is representative of similar results observed in six lesions from six mice. **c**, Representative two-photon in vivo time-lapse images of perilesional astrocytes 1–21 days after 2Phatal VOL ablation in *Aldh1l1*^GFP^ mice. The cyan dashed line indicates the border of the 2Phatal ablated area. The slow disappearance of 2Phatal-targeted astrocytes within 2 weeks was noted. At the last imaging time point, Hoechst 33342 (in white) was added to counterstain for nuclei (astrocytes are shown in red). Stationary (top) and translocating (bottom) daughter-cell nuclei are indicated by yellow arrowheads. Image is representative of similar results observed in six lesions from six mice. **d**,**e**, Vector-based quantification of process length (**d**) and polarization (**e**; UA—*n* = 45 astrocytes from three animals, SC—*n* = 47 cells from three animals, SL—*n* = 56 cells from four animals, VOL—*n* = 59 cells from six animals). ****P < 0.0001; Mann–Whitney test, two sided. Thick black lines represent the median, and fine black dashed lines represent the first and third quartiles. **f**,**g**, The percentage of proliferative (**f**) and repopulating (**g**) astrocytes, respectively, showing an increase with larger ablation volumes (UA—*n* = 5 animals, SC—*n* = 3 animals, SL—*n* = 4 animals, VOL—*n* = 6 animals). *P = 0.0101 (VOL versus UA) and *P = 0.0428 (VOL versus SC) in **f**, *P = 0.0345 and **P = 0.0075 in **g**; Kruskal–Wallis test followed by Dunn’s multiple comparisons test. Data are represented as mean ± s.e.m. SC, single cell; UA, unaffected area; SL, single layer; VOL, volume ablation. Schematic in **a** created in BioRender; Wyss, M. https://BioRender.com/nsnje9t (2026). Source data

The recently developed 2Phatal method successfully ablates cells through programmed cell death^18^. This method was originally designed for the ablation of single cell, such as neurons or astrocytes. Therefore, we adapted it to encompass large cell numbers (Fig. 7a,b). In line with previous studies, targeting single astrocytes with 2Phatal induced slow nuclear pyknosis and the formation of apoptotic bodies leading to cell death within 5–10 days^18^. 2Phatal ablation of a group of three to five astrocytes did not elicit a marked response in surrounding astrocytes compared to control areas (Hoechst staining, but no laser ablation). Next, we performed ablation of astrocytes covering an area comparable to the mean area of AQP4-IgG-mediated astrocyte depletion (40,000 µm^2^). This led to prominent reactivity of perilesional astrocytes, characterized by process elongation and polarization in only one astrocytic layer (~30–40 µm in *z* dimension), but did not result in substantial proliferative astrocyte activity (Fig. 7c–g). However, when 2Phatal was performed on several astrocytic domains in all directions (over 100 µm in *z* dimension), surrounding astrocytes not only became polarized but also proliferated and promoted translocation of daughter-cell nuclei through nucleokinesis (Fig. 7c–g). These results indicate that the extent of astrocyte loss influences the magnitude of astrocyte remodeling and repopulation responses.

### Similar features of astrocyte remodeling are present in human astrocytopathy

**Fig. 8:**
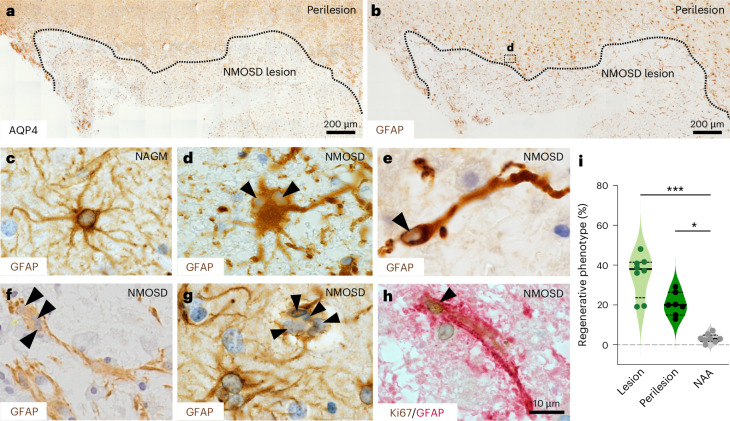
Astrocyte remodeling features in human NMOSD lesions. **a**,**b**, Human brain biopsy sections showing an NMOSD lesion and perilesional area stained for AQP4 (**a**) and GFAP (**b**). Dashed lines indicate lesion border. Boxed area magnified in **d**. **c**, GFAP^+^ astrocyte in NAGM. **d**–**g**, Examples of reactive astrocytes in NMOSD astrocytes stained for GFAP, demonstrating the following: diploid astrocyte (**d**), polarized shape (**e**), triploid cell (**f**) with a leading polarized process and tetraploid cell (**g**). Black arrowheads indicate individual nuclei. **h**, Ki67/GFAP costaining indicating proliferative astrocytes in the perilesional region. **c**–**h**, Scale bar = 10 µm. Nuclear counterstaining with hematoxylin in **a**–**h**. Images are representative of similar results observed in seven human samples. **i**, Percentage of astrocytes within NMOSD lesional and perilesional areas (*n* = 7) compared to NAA (*n* = 8) from seven human samples. *P = 0.0370, ***P < 0.0001; Kruskal–Wallis test. Thick black lines represent the median, and fine black dashed lines represent the first and third quartiles. NAGM, nonaffected gray matter; NAA, nonaffected areas. Source data

Having identified astrocytes with pronounced remodeling features after AQP4-IgG-mediated astrocyte loss in the mouse brain, we then asked whether similar astrocytic features are present in human AQP4-IgG-mediated lesions. For this purpose, we examined human brain tissue from confirmed cases of AQP4-IgG^+^ NMOSD (Extended Data Table 1). Lesions were identified by AQP4 protein loss and by marked GFAP upregulation at lesion borders (Fig. 8a,b). Indeed, a fraction of reactive astrocytes in the perilesional and lesional region showed a multinucleated state combined with elongated and polarized morphologies (Fig. 8d–h), which were significantly more abundant than in nonaffected areas (Fig. 8c,i). Moreover, some perilesional astrocytes were positive for the proliferation marker Ki67 (Fig. 8h). These observations indicate that astrocytes in human AQP4-IgG-mediated pathology can show morphological and proliferative features similar to those observed in the mouse model.

## Discussion

Astrocyte loss is a common feature of brain injury and numerous neurological disorders. However, our understanding of the spatiotemporal dynamics of astrocyte remodeling and repopulation after astrocyte loss in the adult brain remains limited.

Here we developed an AQP4-antibody-based selective and focal astrocyte ablation paradigm that does not induce glial border formation and is compatible with chronic in vivo two-photon microscopy. Using this approach, we characterized how surviving perilesional cortical astrocytes respond to focal loss of astrocytes over time. We observed pronounced structural remodeling, transient changes in calcium signaling and a multinucleated interstage in perilesional astrocytes, accompanied by the translocation of daughter-cell nuclei into previously astrocyte-depleted territories. These cellular responses were associated with a transient transcriptional signature and were strongly influenced by the extent of astrocyte loss. Together, these findings provide a detailed, longitudinal description of astrocyte remodeling and repopulation dynamics after selective and spatially confined astrocyte ablation in the adult brain.

Adult astrogenesis has previously been described in neurogenic niches such as the dentate gyrus^49^ and the subependymal zone^50^, and more recently at low levels in a subset of white matter astrocytes of the corpus callosum^51^, but is rarely found in the cortex under homeostatic condition. However, in response to brain injury, proliferating multinucleated astrocytes have been reported both in rodents and humans^4,7,8,52–54^, but their spatial dynamics and cellular behaviors have remained poorly characterized in vivo. In line with these earlier studies, we detected increased astrocyte proliferative activity across several focal injury paradigms, including astrocytopathy-driven neuroinflammatory lesions, apoptotic astrocyte ablation and stroke. Notably, however, proliferation alone did not predict the extent of astrocyte repopulation, consistent with previous studies showing that astrocyte proliferation does not necessarily lead to restoration of astrocytic territories^9–11^. Our high-resolution in vivo imaging approach allowed us to directly visualize how astrocytes remodel their morphology and reposition daughter-cell nuclei over extended periods. We found that astrocyte repopulation in the absence of glial border formation was associated with sustained multinucleated states and gradual nuclear translocation through shared cytoplasm, rather than with long-range cell migration. This contrasts with developmental neurogenesis, where neural and glial progenitors migrate along radial glial fibers into the cortex^55,56^. In the adult brain, our data are consistent with previous studies that failed to observe directed astrocyte migration in border-forming lesions^7–9^. Instead, astrocyte repopulation after spatially confined astrocyte loss appears to rely on extensive structural remodeling, including polarization, process elongation and centripetal translocation of daughter-cell nuclei toward the lesion core.

Within the CNS, nucleokinesis is a well-characterized component of neural progenitor migration during development, involving coordinated cytoskeletal remodeling and saltatory nuclear movement^26,57^. The real-time visualization of nuclear translocation in adult astrocytes provided here demonstrates that similar cellular machinery can be engaged outside of developmental contexts. In awake mice, we observed nuclear translocation speeds and pause dynamics comparable to those reported for migrating progenitors^26,56–58^. These movements were accompanied by transient nuclear deformation, consistent with the mechanical constraints imposed by nuclear translocation^57^. While the functional significance of these nuclear movements in astrocytes remains to be determined, their consistent association with astrocyte repopulation in border-free lesions suggests that nucleokinesis is a prominent feature of the astrocyte response to focal astrocyte loss.

Astrocyte behavior following astrocyte loss has primarily been studied in traumatic injury, stroke or genetic astrocytopathies such as Alexander disease^4^. In these contexts, astrocytes typically contribute to glial border formation rather than repopulation of the lesion core. Consistent with this, we found that astrocyte repopulation and nuclear translocation were markedly reduced in border-forming stroke lesions of comparable size. By contrast, when astrocyte loss occurred without border formation, in both the AQP4-IgG model and large-volume apoptotic ablation paradigms, astrocyte remodeling and repopulation were more pronounced. The extent of astrocyte loss strongly influenced the magnitude of these responses, suggesting that large-scale astrocyte depletion may exceed the compensatory capacity of hypertrophy and process elongation alone. Although our data do not establish a causal mechanism, they highlight lesion volume as a key parameter shaping astrocyte remodeling dynamics.

Astrocytic contact proteins, such as connexins, Hepacam, neuroligin and AQP4, are known to regulate astrocyte maturation, proliferation and territorial organization^59,60^. Indeed, we observed downregulation of several contact-inhibition-associated genes, including *Hepacam*, *Aqp4* and *Gja1* in perilesional astrocytes after selective astrocyte loss. This transcriptional shift was accompanied by reduced Cx43 expression, yet genetic deletion of astrocytic connexins did not impair astrocyte remodeling or repopulation in our model. These findings suggest that, while contact-inhibition pathways may be altered after astrocyte loss, connexin-mediated gap junction signaling is not required for the observed astrocyte responses. More broadly, our ST analyses revealed a transient injury-associated transcriptional state that resolved over time as astrocyte repopulation progressed. This dynamic regulation aligns with recent studies showing context-dependent astrocyte state transitions after inflammatory or degenerative insults^31,61–63^ and suggests that adult astrocytes retain substantial transcriptional plasticity.

The relevance of these findings to human disease is underscored by our analysis of NMOSD brain tissue. In human AQP4-IgG^+^ lesions, we observed multinucleated, elongated and occasionally proliferative astrocytes in perilesional and lesional regions—features that resemble the astrocyte remodeling responses observed in our mouse model. Similar bipolar GFAP^+^ cells have previously been reported in NMOSD lesions and interpreted as astrocyte precursor-like cells of uncertain origin^64^. Our data suggest that astrocyte remodeling responses involving multinucleation and nucleokinesis may also occur in human astrocytopathies. However, the functional consequences of these cellular states in NMOSD or other neurological diseases remain unknown. Moreover, the focal astrocyte-depletion models used here do not capture the full complexity of human disease pathology, including immune-mediated mechanisms, chronic inflammation and widespread tissue damage.

In conclusion, using multimodal, high-resolution in vivo imaging and STs, we provide a comprehensive characterization of astrocyte remodeling and repopulation after focal astrocyte loss in the adult brain. Our findings reveal that astrocyte responses to focal astrocyte depletion involve sustained multinucleated states, nuclear translocation and dynamic transcriptional changes, with the magnitude of these responses strongly influenced by lesion context and size. While the functional implications of these cellular behaviors remain to be established, this work highlights the remarkable plasticity of mature astrocytes and provides a foundation for future studies investigating how astrocyte remodeling contributes to tissue responses in neurological disease.

## Methods

### Animals

In this study, 4–8-month-old male and female mice were used. All animals were kept in an inverted 12-h light/12-hour dark cycle at temperature-controlled and humidity-controlled husbandry conditions (22–24 °C, 50–60% relative humidity) with unlimited access to food and water. Male and female littermates were allocated equally to the control and experimental groups unless explicitly stated otherwise. All animal experiments were approved by the local veterinary authorities according to the guidelines of the Swiss Animal Protection Law, Veterinary Office, Canton Zurich (Animal Welfare Act of 16 December 2005 and Animal Protection Ordinance of 23 April 2008, licenses ZH217/2020 and ZH181/2024).

To visualize astrocytes, the aldehyde dehydrogenase 1 family member L1 *Aldh1l1*^GFP^ mouse strain (Tg(*Aldh1l1-EGFP)OFC789Gsat/Mmucd*) was obtained from MMRRC. For experiments measuring astrocytic calcium activity, C57BL/6J mice from Charles River were injected with viral constructs to induce GCaMP6s expression in astrocytes (for details, see subsection ‘Injection of genetically encoded calcium indicators’). To trace individual astrocytes, *GLAST*^CreERT2^ x *ROSA26*^tdTomato^ mice were injected once with a low dose of tamoxifen (30 mg kg^−1^ intraperitoneal, 3 mg ml^−1^ in corn oil) to obtain sparse red astrocyte labeling. In addition, ssAAV-PHP.eB/2-hGFAP-EGFP-WPRE-hGHp(A) virus (50 µl of 2.0 × 10^13^ particles per ml; Viral Vector Facility, UZH) was administered intravenously to obtain widespread astrocytic GFP expression for identification of the perilesional area.

*GLAST*^CreERT2^ x *Cx30/Cx43*^fl/fl^ mice^46^ were used to study the role of astrocytic gap junction coupling 4 weeks after tamoxifen injection (for five consecutive days, 100 mg kg^−1^ intraperitoneal, 10 mg ml^−1^ in corn oil). Littermates negative for Cre served as control mice (*GLAST*^+/+^ x *Cx30/Cx43*^fl/fl^). Over 90% loss of Cx30 and Cx43 in the cortex has been previously confirmed in immunohistochemical sections of these knockout mice at 30 dpi^46^. The ssAAV-PHP.eB/2-hGFAP-EGFP-WPRE-hGHp(A) was administered intravenously injected (50 µl of 2.0 × 10^13^ particles per ml) 3 weeks before surgery for visualization of astrocytes. To track nuclei in vivo, the *GLAST*^CreERT2^ x *Ai75D* mice were injected with tamoxifen (for five consecutive days, 100 mg kg^−1^ intraperitoneal, 10 mg ml^−1^ in corn oil) 4 weeks and with ssAAV-PHP.eB/2-hGFAP-EGFP-WPRE-hGHp(A) for visualization of astrocytes (50 µl of 2.0 × 10^13^ particles per ml) 3 weeks before surgery.

### Surgical interventions

Surgical interventions were performed on two separate days. During anesthesia, vitamin A eye ointment was applied to prevent corneal desiccation. To prevent dehydration, 10 ml kg^−1^ of prewarmed Ringerfundin was subcutaneously injected before beginning the intervention. Oxygen was delivered (200 cc min^−1^) to prevent hypoxemia. Animals’ vital signs and temperature were monitored and controlled through MARTA Pad (Vigilitech).

#### Headplate implantation

Animals were anesthetized with isoflurane (2–2.5%) in a mixture of O_2_ and air (30%/70%) at a flow rate of 300 ml min^−1^. For headplate implantation, animals were fixed in a stereotaxic frame (Model 900; David Kopf Instruments). The head region was shaved, disinfected (Kodan; Schulke & Mayr), and anesthetized by local subcutaneous injection of a mixture of lidocaine (10 mg ml^−1^) and bupivacaine (5 mg ml^−1^). The skull surface was exposed by a midline incision (1.2–1.5 cm long), cleaned by removing the connective tissue and covered with a bonding agent (One Coat 7 Universal, Coltene). Next, a custom-made stainless steel headplate was positioned centrally above the exposed bone and attached by multiple layers of blue light-curing dental cement (Tetric EvoFlow, Ivoclar Vivodent) while sparing the area designated for later craniotomy.

#### AQP4-IgG/Ctrl-IgG injection

Next, 2–3 days after headplate implantation, animals were anesthetized by intraperitoneal injection of a mixture of fentanyl (0.05 mg kg^−1^ bodyweight (BW); Sintenyl; Sintetica), midazolam (5 mg kg^−1^ BW; Roche) and medetomidine (0.5 mg kg^−1^ BW; Orion Pharma) and reapplied as needed (after about 60 min). A craniotomy above the left somatosensory cortex was performed using a dental drill (0.2-mm diameter, H-4-002HP; Rotatec GmbH). After craniotomy and durotomy, a mixture of a human IgG1 recombinant antibody AQP4-IgG (0.1–3 µg µl^−1^, clone 7-5-53), reconstructed from a clonotypic plasma blast obtained from the cerebrospinal fluid of a patient with AQP4-IgG^+^ NMOSD^65^ together with human complement (15–30 U ml^−1^; Sigma-Aldrich, S1764), was injected using a custom-made microinjector and a pulled glass capillary (Drummond PCR micropipettes, Drummond Scientific) with a tip diameter of 35–45 µm. A volume of 130 nl was distributed between 0 and 300 µm depth in 100 µm steps (Fig. 1a), and additional 90 nl was slowly infused (20 nl min^−1^) during insertion and retraction of the pipette to keep it patent. In control lesions, the recombinant Ctrl-IgG (0.1–3 µg µl^−1^, clone ICOS-5-2) of the same isotype as AQP4-IgG (human IgG1), of unknown specificity from a patient with meningitis, was injected together with complement in the same fashion^65^. For two-photon imaging, a chronic window was implanted (see subsection ‘Chronic window implantation’). Anesthesia was reversed by intraperitoneal injection of a mixture of flumazenil (0.9 mg kg^−1^ BW) and atipamezole (45 mg kg^−1^ BW). After surgeries, animals were treated with buprenorphine (0.1 mg kg^−1^ BW, subcutaneous) and carprofen (10 mg kg^−1^ BW, subcutaneous). Thereafter, carprofen treatment was continued twice a day for 3 days.

#### Injection of genetically encoded calcium indicators

In C57BL/6J mice, headplate implantation, craniotomy and durotomy were performed as described above. Three to four neighboring injections of adeno-associated virus (1.6 × 10^12^ particles per ml of AAV9-hGFAP-GCaMP6s; Viral Vector Facility, UZH; 125 nl per injection) were performed with a custom-made microinjector to achieve multiple overlapping spots where astrocyte depletion was later induced (see above). Viral injections were performed through a glass capillary at a depth of 350 and 200 μm from the brain surface. Large blood vessels were avoided to prevent bleeding and hemoglobin-mediated light absorption during imaging. A chronic window was implanted (see subsection ‘Chronic window implantation’). Three weeks after virus injection and baseline imaging, the chronic window was again removed and AQP4-IgG/Ctrl-IgG/complement was injected.

#### Chronic window implantation

For chronic two-photon measurements, a square sapphire glass (3 × 3 mm; HEBO Spezialglas) was implanted after the completed virus or antibody injection. The glass was gently placed and sealed with light-curing dental cement (Tetric Evoflow, Ivoclar Vivodent).

#### In vivo nuclear staining

In a subset of animals, the cranial window was removed 30 days after lesion induction and the nuclear dye Hoechst 33342 (0.1 mg ml^−1^, 2 × 15 min) was applied for nuclear staining in vivo. After implantation of a new chronic window, lesions were imaged the following day.

### Behavior training for awake two-photon imaging

Mouse handling started before headplate implantation, multiple times a day for 3 days, to familiarize the animals with the experimenter. Two days after headplate implantation, handling was resumed. Animals were adapted to the head fixation by restraining them with the implanted headplate several times a day, with a gradual increase in restraint time from seconds up to several minutes. The last few training sessions took place in the microscopic setup with the microscope and shutters operating to familiarize the animals with the setup. After an extensive training period of about 1 week, animals tolerated head fixation for the duration of a 15–20 min imaging session while being able to move on a custom-made air-lifted platform.

### Anesthetized and awake in vivo two-photon microscopy imaging

Two-photon imaging was performed with a custom-made two-photon laser scanning microscope^66^ equipped with two two-photon lasers (Chameleon Discovery NX, Coherent) and a ×25 water-immersion objective (W Plan-Apochromat ×25/1.05 NA, Olympus). The tunable laser was set to 920 nm to excite GFP and GCaMP6s or to 780 nm to excite Hoechst. A second laser with a fixed 1,040 nm wavelength line was used to excite tdTomato. The emitted light was focused on the photomultiplier (Hamamatsu, H9305-03) equipped with emission filters for blue fluorescence (475/64 Brightline HC, AHF Analysentechnik), green (535/50 Brightline HC, AHF Analysentechnik) and red fluorescence (607/70, 607/70 Brightline HC, AHF Analysentechnik). A dichroic mirror at 506 and 560 nm separated the emission light beam. ScanImage^67^ and custom-written LabVIEW software were used for image control and data acquisition.

#### Anesthetized imaging

Mice were anesthetized using isoflurane. During image acquisition, isoflurane was supplied through a face mask (1.5–2% in O_2_ and air). Thus, 24 h after antibody injection, the first images were acquired and then afterwards at predefined time points (see Results). Stacks were acquired with 512 × 512 pixels at 0.74 Hz for overview and with 2,048 × 2,048 pixels at 0.37 Hz for detailed signal analysis. For calcium measurements, we chose a concentration of isoflurane that has previously shown very little interference with baseline calcium activity in astrocytes^68^. Calcium signals were measured at frequencies of 11.84 Hz over a period of 120 s (128 × 128 pixels, 0.5 ms per line, zoom 6) from two to three different spots of astrocyte-depleted or control lesions.

#### Vasculature imaging

The vasculature was labeled by administered intravenous injection of 50 μl of 2.5% (wt/vol in saline) of 70 kD Texas Red dextran (Thermo Fisher Scientific, D1830). The dye was excited at a wavelength of 870 nm. For structural assessment of the capillary bed, *z* stacks were acquired at 1.0-μm step size with a resolution of 512 × 512 pixels at 0.74 Hz. Red blood cell velocity was measured along the vessel midline with line scans (256 × 256 pixels, 6.1 Hz) and subsequently processed by a custom-designed image processing toolbox (Cellular and Hemodynamics Processing Suite^69^ in MATLAB (The MathWorks, R2017b)) using the Radon transform. Using a Gaussian fitted intensity profile drawn perpendicular to the vessel midline, the vessel’s diameter was calculated at full-width half maximum.

#### Awake imaging

Twenty-four hours after antibody injection, initial images were acquired. After 3–5 days, acquisitions were performed every 3 h for six consecutive days, followed by imaging sessions at lower frequency as for anesthetized imaging (2, 4 and 8 weeks).

### Photothrombotic stroke induction

The photosensitive dye Rose Bengal (4,5,6,7-tetrachloro-2′,4′,5′,7′-tetraiodofluorescein; 100 µl administered intravenous, 20 mg ml^−1^ in NaCl; Thermo Fisher Scientific, A17053) was administered through tail vein catheter. After established protocols^70^, feeding and draining arterioles and venules within a ~400 µm radius field were targeted using a two-photon laser line scan (720 nm, 90 mW power at the objective) along the vessel midline until complete cessation of blood flow. Photoactivation of the dye generates reactive oxygen species finally leading to thrombosis. To generate a localized, circumferential lesion, multiple feeding and draining arterioles were irradiated and occluded to disrupt blood supply to the central vascular territory of interest.

### 2Phatal astrocyte ablation

Headplate implantation, craniotomy and durotomy were performed in *Aldh1l1*^GFP^ animals as described above. Hoechst 33342 (0.1 mg ml^−1^; Invitrogen) was a bath applied topically for 2 × 5 min. Hoechst was thoroughly washed from the cortical surface with Ringer’s solution and a cranial window was put in place as described above. One day after surgery, fluorescently labeled nuclei of different numbers of astrocytes were targeted over a square region of interest (ROI) with a focused two-photon laser (Chameleon Discovery NX, Coherent) tuned to a wavelength of 775 nm. ROIs were scanned 240 times with 16 × 16 pixels at 23.67 Hz (resulting in 10 s total irradiation time) through a ×25 water-immersion objective (W Plan-Apochromat ×25/1.05 NA, Olympus) at 50 mW laser power (measured under the objective) to induce apoptosis (as described in ref. ^18^). Laser output power was increased by 10 mW for every 30 µm increase in *z* direction for cells in deeper areas to account for light scattering and absorption. Images of the lesion area were acquired under isoflurane anesthesia at different time points after 2Phatal cell ablation.

### Image analysis

Images were processed using the open-source image analysis software Fiji (ImageJ (v2.1.0))^71^. For display purposes, two-dimensional (2D) images were generated using *z*-maximum intensity projections for merged channels. In nonquantitative panels, the γ value was adjusted nonlinearly to enhance visibility of low-intensity objects. Datasets were processed with Excel (Microsoft). Figures 1–8 and Extended Data Figs. 1–9 were crafted with Affinity Designer 2 (Serif). Graphical illustrations of experimental protocols were generated with BioRender.

#### Vector analysis

Astrocytes were chosen randomly from perilesional areas. Distal ends of primary astrocytic processes, location of cell soma, as well as the center of the lesion were manually marked by single-point ROIs in two-photon image stacks taken 1, 5, 14 and 60 days after AQP4-IgG-mediated lesion induction. Coordinates of ROIs in *x*, *y* and *z* were extracted. From these coordinates, every process was assigned a vector that reflected its length and orientation. This allowed for calculation of an average vector for every cell (equation (1,1)) by means of a custom-written Python script (v3.6.5), summarizing both length and orientation of the cell’s primary processes. These measures could then be compared over different time points. In controls, the average diameter of AQP4-IgG-mediated lesions at 1 dpi was used as a safety margin to avoid analyzing possible astrocytic changes in close proximity to mechanical injury. Only astrocytes outside of, but adjacent to, this margin were quantified.

1 $${\bf{U}}=\,\frac{1}{n}\,\mathop{\sum }\limits_{i=1}^{n}\left(\begin{array}{l}{x}_{i}\\ {y}_{i}\\ {z}_{i}\end{array}\right)\,$$

Here the coordinates of the average vector **U** are calculated by averaging the *x*, *y* and *z* coordinates of all vectors within a cell.

#### Lesion size and cell number analysis

In vivo image stacks of *Aldh1l1*^GFP^ mice taken 1 day after antibody injection were *z* projected over 50–150-µm depth and used for lesion area calculation. The number of intralesional astrocytes was assessed by manually counting all GFP^+^ cell bodies that appeared within this lesion area over different imaging time points.

#### Proliferation rate

All perilesional astrocytes of a randomly chosen quadrant of the lesion were marked in images taken 1 dpi and then tracked over several time points (up to 60 dpi) to detect proliferation. In control conditions, astrocytes at the same average distance from the site of injection were tracked similarly in random quadrants.

#### Calcium activity

Image analysis was performed using ImageJ and a custom-designed image processing toolbox, Cellular and Hemodynamic Image Processing Suite^69^ based on MATLAB (The MathWorks, R2017b). Calcium transients were quantified by an unbiased algorithm^23,72^. ROIs were selected by combining hand selection of astrocytes based on the high-resolution acquisition (512 × 512 pixels) and automated ROI detection with an activity-based algorithm^72^ using the 128 × 128 pixel images. A 2D spatial Gaussian filter (*σ* = 2 µm) and a temporal moving average filter (width = 1 s) were applied to all images to reduce noise. A moving threshold for each pixel was defined in the filtered stack as the mean intensity, as well as five times the s.d. of the same pixel in the preceding 3 s. Using this sliding box-car approach, active pixels were identified as those that exceeded the threshold. Active pixels were grouped in space (radius = 2 µm) and time (width = 1 s).

#### Astrocyte territory analysis

Territories of sparsely labeled tdTomato^+^ astrocytes in in vivo images were segmented using the Fiji segmentation editor plugin on multiple optical sections of the *z* stacks. ROIs were interpolated across slices, and the ROI area was determined for every slice. Astrocyte territories were compared between images acquired at 1 dpi and 5 dpi. The 3D surface rendering and visualization were performed using Imaris software (v10.0; Bitplane, Oxford Instruments).

#### Nuclear shape factor calculation

DAPI stainings in fixed sections from AQP4-IgG and Ctrl-IgG-injected areas at 5 dpi were analyzed. All astrocytes within the lesion were included and the shape factor (length-to-width ratio) of their nucleus was calculated for each cell.

#### Interventional experiments

In connexin knockout mice, lesion size, vector-based process length, orientation and proliferation rate were analyzed between genotypes in a blinded manner.

### Vessel segmentation analysis

Three-dimensional volumes were segmented by slicing the volume along the depth direction into 2D slices. To avoid bias in the segmentation algorithm toward extreme values, we set all values >99th percentile intensity to this cutoff value. To obtain the threshold value for segmentation, we used Li pattern recognition^73^, implemented in the Python scikit-image library^74^. To prevent extreme threshold values of the Li algorithm in the presence of imaging artifacts, we set the segmentation threshold to the mean value of the 3D volume if the obtained threshold is less than this for the respective 2D slice. To remove noise and small pixel clusters, one binary opening operation was performed after thresholding. To filter out superficial (depth = 0–20 μm) large vessels from the 3D volumes, connected areas that covered more than 200,000 voxels were disregarded. The vessel volume was calculated by multiplying the number of voxels classified as vessels by the physical volume of a voxel. The volume fraction was calculated by dividing the number of voxels classified as vessels by the total number of voxels in the ROI. To obtain the vasculature surface area, we used the findContours function of OpenCV^75^. The vessel diameter was calculated by skeletonizing the segmentation^76^ and applying a median filter of voxel size three. After sampling points along the centerline and calculating the distance between each point and the inverse segmentation, the minimum calculated distance was used as the radius of a vessel at a respective point.

### Immunohistochemistry and histological analysis

Animals were intraperitoneally injected with pentobarbital (200 mg kg^−1^ BW) for terminal anesthesia. Animals were perfused transcardially with 4% paraformaldehyde (PFA) in 0.0.1 M PBS (1× PBS; 1.5 mM KH_2_PO_4_, 2.7 mM KCl, 8.1 mM Na_2_HPO_4_ and 137 mM NaCl), postfixed for 3 h in 4% PFA, then dissected and transferred to 30% sucrose (Sigma-Aldrich) solution in PBS for cryoprotection overnight at 8 °C. Brains were placed in embedding medium (Richard-Allen Scientific, NEG-50) with the apical cortical surface facing the cutting plane and then cut into 30-μm sections in a cryostat (Leica Biosystems AG, CM3050S). Antibodies were diluted in 0.3% Triton X-100 (Sigma-Aldrich) and 5% normal donkey serum (Abcam, ab7475) in Tris–buffered saline. The following antibodies have been used: chicken anti-GFAP (1:1,000; Abcam, ab4674), rabbit anti-Ki67 (1:500; Abcam, ab16667), rabbit anti-SOX2 (1:500; Thermo Fisher Scientific, PA1-094), chicken antivimentin (1:100; Abcam, ab24525), rabbit anti-NeuN (1:700; Abcam, ab177487), rabbit anti-Cx43 (1:300; Cell Signaling Technology, 3512) and rabbit antinestin (1:200; Abcam, ab221660). For staining with nestin, antigen retrieval was performed by microwave for 10 min in 80 °C citric acid (0.1 M citric acid, 0.1 M tripotassium citrate-2-hydrate in ddH_2_O). For all stainings, slices were incubated with the appropriate secondary antibodies and DAPI (1:1,000; Abcam, ab228549) for nuclear counterstaining.

#### Confocal microscopy

Samples were scanned with Zeiss confocal microscopes (CLSM 700, CLSM 800 and CLSM 900) equipped with ZEN 2011 (Black Edition, V7.1) using ×10 air objectives (Plan-Apochromat ×10/0.45 M27, Zeiss), ×25 oil-immersed objectives (LCI Plan-Neofluar ×25/0.8 Imm Corr DIC M27, Zeiss) or ×40 oil-immersed objectives (Plan-Apochromat ×40/1.4 Oil DIC M27, Zeiss). For GFAP, vimentin, nestin and SOX2 quantification, intensity in *z*-projected 30-μm stacks was measured in an area encompassing perilesional astrocytes after subtraction of background intensity in an adjacent unaffected region. The same area size and distance from the lesion core were used for the control condition.

### EdU administration and imaging

EdU was administered through drinking water (0.4 mg ml^−1^) immediately after antibody injection. In addition, a single intraperitoneal injection of EdU (5 mg ml^−1^, 25 mg kg^−1^ BW) in NaCl was given. EdU-supplied water was provided for 7 days and then substituted for regular drinking water. EdU-supplied water was renewed at the latest after 3 days. Animals were perfused 14 days after lesion induction. Detection of EdU incorporation was performed using a commercially available detection kit (BCK-EdU-647, Click-iT, BaseClick). After the detection protocol, either additional stainings were performed as described above or DAPI alone in Tris–Triton buffer was added and left to incubate for 20 min.

### Immunohistochemistry of human biopsy and autopsy tissue samples and image acquisition

Formalin-fixed paraffin-embedded human CNS material was obtained from the archive of the Department of Neuropathology, University Medical Center Gottingen, in accordance with protocol approval by the Ethics Committee of the University Medical Center Gottingen (vote 19/9/10). A positive AQP4-antibody status was confirmed for the patients diagnosed after 2005; the status for one patient autopsied in 1962 is unknown. For this patient, only clinical data are available, which indicate that the patient presented with acute myelitis and area postrema syndrome, both key features of AQP4-IgG^+^ NMOSD. The diagnosis was histologically and immunohistochemically confirmed by the observation of lesions with damaged astrocytes, the loss of GFAP and AQP4, as well as variable demyelination and macrophage infiltration.

For our present study, 5–6-µm-thick slices were cut. Nuclear structures were labeled using hematoxylin. Immunohistochemical stainings were performed against GFAP (1:50; Dako, M0761) and AQP4 (1:200; Sigma-Aldrich, A5971). Proliferating cells were nuclear labeled with the Ki67 antibody (1:200; Dako, GA626). The primary antibody binding was visualized using a biotinylated secondary antibody, followed by developing with avidin-peroxidase and diaminobenzidine or 3-amino-9-ethylcarbazole.

Whole slide scans of GFAP-stained tissue slices were acquired at ×200 magnification using an Olympus VS120 slide scanner for quantification of multinucleated astrocytes. To reveal morphological features of single cell, high-magnification (×400, ×1,000) images of selected regions were acquired using an Olympus BX63 microscope. For astrocyte phenotype quantification, cells were classified as ‘migratory progenitor-like astrocytes’ only if they showed multinucleation and/or a bipolar morphology.

### Correlative light and electron microscopy

#### Section preparation

Animals were intraperitoneally injected with pentobarbital (200 mg kg^−1^ BW) for terminal anesthesia. Mice were perfused with a fixative containing 2% formaldehyde and 2.5% glutaraldehyde in phosphate buffer (0.1 M, pH 7.4) after the final imaging session. Brains were removed from the skull and postfixed in the same fixation solution for 3 h, washed briefly in phosphate buffer and 100-µm-thick sections were collected with a vibratome (Leica VT1200 S). Sections were stained with nuclear staining DAPI (1:500) for 20 min, followed by imaging with a multiphoton microscope (Leica SP8 MP Dive Falcon), equipped with a ×25 NA 1.00 water objective (HC IRAPO L ×25/1.0 W motCORR; Leica Microsystems). We acquired three fluorescence channels (excitation laser at 720 nm DAPI; excitation laser at 950 nm eGFP; excitation laser at 1,040 nm tdTomato) and used a transmission PMT to obtain additional structural information (location of blood vessels). Overview images of the whole sections were collected as well as complete *z* stacks of ROIs (voxel size = 170 × 170 × 800 nm). The sections were placed back into fixative solution before further processing for electron microscopy.

#### Serial-section electron microscopy

The vibratome sections were fixed with 1% OsO_4_ for 1 h in 0.1 M cacodylate buffer at 0 °C, and in 1% aqueous uranyl acetate for 1 h at 4 °C. Samples were dehydrated in an ethanol series and embedded in Araldite/Epon (Sigma-Aldrich) 66% in propylene oxide overnight, 100% for 1 h at room temperature and polymerized at 60 °C for 20 h. The embedded samples were correlated with the transmission light microscope images acquired with the two-photon microscope to trim the ROI before the serial sectioning. Ultrathin serial sections (100 nm) were collected on silicon wafers using an ultramicrotome (Artos 3D; Leica Microsystems) equipped with a silicon wafer holder (CD-FH^77^). Specimen sections on silicon wafers were poststained using Reynolds lead citrate. Sections were imaged in an Apreo 2 VS scanning electron microscope using the MAPS software package for automatic serial-section recognition and image acquisition^78^ (Thermo Fisher Scientific). The following parameters were used for imaging: OptiPlan mode, T1 detector; pixel size of 4 nm or 7 nm, pixel dwell time of 3 or 5 µs, an electron high tension of 1.8 keV and a beam current of 0.1 nA. Serial-section TIFF images were aligned and the pairs of cells of interest were traced using the Fiji^71^-plugin TrakEM2 (ref. ^79^). Traces and aligned images were imported for 3D reconstruction in Imaris (Oxford Instruments).

### Statistics and reproducibility

All experiments in this study include at least three biological replicates, except for the ST analysis at time points of 5 dpi and 17 dpi (four lesions from two animals, respectively). The number of biological replicates (*n*) is indicated in the legends of Figs. 1–8 and Extended Data Figs. 1–9. No statistical methods were used to predetermine sample size, but our sample sizes are similar to those reported in previous in vivo imaging publications^20,21^. Randomization was not performed for this study as there was no particular intervention (for example, treatment) planned. Whenever possible, littermate controls were used, and an equal number of mice were allocated to different groups within one cage to rule out cage effects. Analyses were performed on randomly selected datasets, as specified in the respective sections. Data collection and analysis were performed in a blinded manner, unless this was not possible due to an obvious disease or labeling phenotype. Based on pre-established exclusion criteria (based on the animal protocol), animals showing signs of traumatic damage to the brain during craniotomy or bleeding were excluded from the study. Structures with an insufficient signal-to-noise ratio during in vivo imaging were also excluded. Tissue with insufficient labeling or staining was not analyzed. As non-normal data distribution was assumed for all analyses, formal testing for normality and equal variances was not performed, and nonparametric statistical methods were applied throughout. Statistical analysis was performed in GraphPad Prism 9 and 10 software. Data are represented as mean ± s.e.m. Statistical significance was calculated using the Mann–Whitney test for comparing two unpaired groups and nonparametric analysis of variance followed by the Kruskal–Wallis test for comparing more than two groups. Statistical comparison of two paired groups was calculated by a nonparametric Wilcoxon signed-rank test. Comparisons of more than two paired groups were performed using a nonparametric Friedman test followed by Dunn’s multiple comparisons test. Correlation analysis was performed with a nonparametric Spearman’s correlation. *P* values of <0.05 were considered statistically significant and indicated with asterisks in graphs as *P < 0.05, **P < 0.01, ***P < 0.001 and ***P < 0.0001.

### STs

#### Tissue processing

Somatosensory cortices from *Aldh1l1*^GFP^ mice predominantly containing two lesions were collected at three distinct time points postinjection—3 dpi (*n* = 4), 5 dpi (*n* = 2) and 17 dpi (*n* = 2). At each time point, animals were perfused with 4% PFA supplemented with 10% formalin, followed by brain extraction and postfixation in the same solution for 24 h. Brains were then transferred into 70% ethanol for at least 24 h before dehydration and embedding in paraffin wax. Embedded tissues were sectioned at 5-μm thickness using a microtome with a water bath maintained at 40 °C, with individual sections placed onto separate slides. Upon reaching lesion boundaries, residual tissue was assessed for RNA quality, with all selected samples showing DV200 values above 60%. Before sequencing, slides were deparaffinized and decrosslinked, followed by immunostaining. Tissue sections were incubated with primary antibodies targeting goat anti-Iba1 (1:700; Abcam, ab5076) and rabbit anti-NeuN (1:700; Abcam, AB177487). Secondary antibodies included CY5-conjugated antigoat (1:700; Jackson ImmunoResearch, 705-175-147), CY3-conjugated antirabbit (1:700; Jackson ImmunoResearch, 711-165-152) and nuclei counterstained with DAPI (1:3,000; Abcam, AB228549). For each mouse, one section was selected based on the RNA integrity and confocal microscopy evaluation. All experimental steps were conducted according to the Visium HD Spatial Gene Expression Reagent Kits User Guide (10x Genomics, CG000684).

#### Library preparation and sequencing

Libraries were prepared according to the Visium HD Spatial Gene Expression protocol (10x Genomics). Sequencing was performed at the Functional Genomics Center Zurich on an Illumina NovaSeq X Plus system using 150 bp paired-end reads (150:10:10:150) across two lanes of a 10B flow cell. An average of 1,648,804,428.13 reads per sample were generated, detecting approximately 16,600.75 genes per sample with an average sequencing saturation of 80.45%. Reads were aligned to the mouse reference genome (GRCm39) using Space Ranger (v3.1.3).

#### Data processing

ST data were processed using an 8 × 8 μm binning, as recommended by 10x Genomics. Analyses were conducted in R (v4.4.2) using Seurat (v5.2.1). ROIs were delineated by manually annotating lesion centers in confocal tissue images. Spots located within an 800-μm radius of annotated lesion centers were retained, a distance chosen to minimize overlap across adjacent lesions. Distances between astrocytic coordinates and annotated lesion centers were computed in 2D space using Euclidean distance. Next, quality control was performed before downstream analysis by excluding outliers using SpotSweeper^80^ and removing spots containing <100 unique molecular identifiers according to the inflection point in the unique molecular identifier distribution. Postfiltering, spot-level counts were normalized with SCTransform (SCT)^81^. All downstream analyses—including clustering, differential expression (DE) and gene signature scoring—were performed using SCT-normalized data and Pearson residuals derived from the SCT model. Samples collected at the same time point were integrated using the SCT-based integration pipeline, with 2,500 highly variable genes selected through the Seurat function SelectIntegrationFeatures for downstream analysis. Dimensionality reduction was performed using principal component analysis, using the first 20 principal components. Graph-based clustering was conducted using the Louvain algorithm implemented in Seurat. A resolution of 0.5 was selected, resulting in 16 clusters at 3 dpi, 16 clusters at 5 dpi and 17 clusters at 17 dpi.

#### Cell-type identification

Cell-type annotation was conducted using MapMyCells^82^, through hierarchical correlation mapping against the 10x Whole Mouse Brain Taxonomy (CCN20230722). Clusters were assigned cell identities based on the proportion of cell types per cluster, mapping confidence probabilities and verification of established cell-type-specific marker gene expression. Cell identities were validated through marker gene expression analysis using Seurat’s FindMarkers function, applied to SCT-normalized data (parameters: min.pct = 0.25, logfc.threshold = 0.25) and further confirmed by confocal imaging. We further filtered astrocyte-specific clusters using the following rigorous procedure: spots expressing at least one astrocyte marker (Gfap, S100b, Aqp4, Aldh1l1, Slc1a2, Gja1, Tubb2b) were retained, while those expressing two or more markers indicative of microglia, vascular cells or oligodendrocytes were excluded. An additional filter was applied to remove spots where PLLP expression was at least twofold higher than either Gfap or Aqp4, further reducing potential oligodendrocyte contamination. These filtering steps excluded up to 20% spots per time point, resulting in final astrocyte spot counts of 364,577 at 3 dpi, 274,169 at 5 dpi and 313,884 at 17 dpi. Astrocyte subcluster-specific marker genes were compared with published healthy and reactive astrocyte panels from different laboratories, including models of lipopolysaccharide-induced inflammation, ischemic stroke^30,33,35^, experimental autoimmune encephalomyelitis^31,34^ and the meta-analytic O’Shea panel^32^. Significant enrichment of overlapping genes was assessed using hypergeometric tests with Benjamini–Hochberg correction. We then quantified overlap between panels using an UpSet representation and derived new ‘consensus’ gene sets based on genes present in five, four, three or two panels, followed by the hypergeometric enrichment analysis. Overall, the pairwise and higher-order overlaps were modest, indicating that most panels capture only partially overlapping aspects of astrocyte reactivity. Next, we grouped genes by how many panels they appeared in and built four new overlap-derived gene sets—genes present in ≥5 panels (14 genes), genes present in ≥4 panels (35 genes), genes present in ≥3 panels (99 genes) and genes present in ≥2 panels (315 genes). We then tested whether each of these overlap bins was enriched among the 3 dpi upregulated injury-associated DEGs using the hypergeometric framework. This analysis showed significant enrichment for the upregulated direction in the ≥2**-**panel gene sets, indicating that the most consistently recurrent genes across independent datasets are strongly overrepresented among our injury-associated astrocyte signature.

#### Gene expression and pathway analysis

Differential gene expression analysis between injury-associated and unaffected astrocytes was performed using Seurat’s FindMarkers function applied to SCT-normalized data. Statistical comparisons were based on Pearson residuals from the SCT model, as this approach offers variance stabilization, particularly suited for small and heterogeneous cell populations^81^. In this spatial analysis, astrocyte transcriptomic profiles gradually transition depending on their spatial proximity to the lesion core, rather than forming sharply distinct groups. To mitigate the risk of false-positive results in DE analyses, particularly for genes showing extreme fold-change values, we used an MA-plot-based filtering strategy. Specifically, genes identified as having low mean expression (bottom, tenth percentile) coupled with high fold-change values (top, tenth percentile) were flagged as potentially noise driven and excluded from subsequent analyses. Identified genes of interest were subsequently assessed across multiple dimensions, including their expression across conditions and time points, as well as their spatial distribution relative to the lesion center.

DE testing was performed using the Wilcoxon rank-sum test. To correct for multiple hypothesis testing, *P* values were adjusted using the Benjamini–Hochberg method for multiple comparisons (parameters: min.pct = 0.1, logfc_threshold = 0.25), with an adjusted *P* value threshold of 0.05. DEGs identified at the earliest time point (3 dpi) were longitudinally assessed at subsequent time points (5 and 17 dpi) by examining overall expression and log fold changes relative to unaffected astrocytes. Functional enrichment analyses were performed using clusterProfiler package^83^ in R and the org.Mm.eg.db annotation database, focusing on GO Biological Process (GO:BP) categories. Temporal changes in enriched pathways were analyzed across all time points. To analyze gene expression as a function of lesion proximity, the previously calculated distances were used. Mean expression levels of individual genes, as well as aggregated migratory and proliferative signatures, were then assessed in relation to their distance from the lesion center. Mean expression of individual genes was computed per bin (geq2 spots per bin). Data visualization and figure generation were performed using mainly the ggplot2 (ref. ^84^), ComplexHeatmap^85^ and circlize^83^ packages in R.

#### Injury-associated gene signatures

To further characterize the transcriptional signature of perilesional astrocytes during lesion recovery, we then analyzed DEGs associated with cell remodeling and gliogenesis states. For this analysis, we curated gene sets based on gene set enrichment analysis, GO pathway analysis and relevant literature sources. The remodeling signature comprised 18 genes associated with cytoskeletal architecture changes, while the regeneration signature included 32 genes linked to cell-cycle processes and cellular plasticity (Supplementary Table 1). For each time point (3, 5 and 17 dpi), per-cell signature genes were computed by averaging SCT-residual expression across these genes.

#### Astrocyte spatial gene expression

To quantify average gene expression in astrocytes as a function of distance from lesion centers, spatial coordinates of astrocyte spots and lesion center positions were extracted from Seurat objects at each time point. Relative coordinates were calculated by subtracting lesion center centroids from spot coordinates. Distances were then scaled and binned into 30 µm intervals by rounding to the nearest bin. For each individual gene of interest, mean expression per spatial bin was computed across all spots within that bin. These per-spot signature scores were aggregated by spatial bins (retaining bins with *≥*2 spots) to derive average signature expression profiles along the lesion-centric gradient.

### Reporting summary

Further information on research design is available in the Nature Portfolio Reporting Summary linked to this article.

## Online content

Any methods, additional references, Nature Portfolio reporting summaries, source data, extended data, supplementary information, acknowledgements, peer review information; details of author contributions and competing interests; and statements of data and code availability are available at 10.1038/s41593-026-02354-5.

## Supplementary information

Supplementary Information Supplementary Table 1.

Reporting Summary

Supplementary Video 1 Perilesional astrocytes show a transient calcium hyperactivity. Time lapses of GCAMP6s of a perilesional astrocyte (shown in Fig. 2h) at day 5 after AQP4-IgG injection (left) compared to astrocytes from a Ctrl-IgG-injected area (right); time in min:s format (scale bar = 20 µm).

Supplementary Video 2 Stack of a multinucleated astrocyte in vivo. Animated *z* stack (104 µm) of a perilesional astrocyte 30 dpi (4-cell clone) is shown in Fig. 3d. TdTomato^+^ astrocyte (red) with multiple nuclei (yellow arrowheads). Nuclei stained with Hoechst 33342 (cyan) in vivo.

Supplementary Video 3 Awake two-photon time lapses of a perilesional astrocyte. The mature astrocyte from Fig. 4f (red arrowhead) undergoes extensive cytoskeletal remodeling and gives birth to two daughter cell nuclei—one stays in close proximity to the parent cell (yellow arrowhead) and the other is translocated through nucleokinesis to a new territory (cyan arrowhead); time in hours (scale bar = 20 µm).

## Source data

Source Data Fig. 1 Statistical source data.

Source Data Fig. 2 Statistical source data.

Source Data Fig. 3 Statistical source data.

Source Data Fig. 4 Statistical source data.

Source Data Fig. 5 Statistical source data.

Source Data Fig. 6 Statistical source data.

Source Data Fig. 7 Statistical source data.

Source Data Fig. 8 Statistical source data.

Source Data Extended Data Fig. 3 Statistical source data.

Source Data Extended Data Fig. 6 Statistical source data.

Source Data Extended Data Fig. 7 Statistical source data.

Source Data Extended Data Fig. 8 Statistical source data.

Source Data Extended Data Fig. 9 Statistical source data.

## Data Availability

Authors confirm that all relevant data are included in the paper/or its supplementary information files. Further information and requests for resources should be directed to the lead contact, B.W. This study did not generate new unique reagents. Mouse lines can be requested from the providing investigators and are protected by standard MTAs. Stained human tissue sections, whole slide scans thereof and data related to human tissue analysis will be provided upon reasonable request. Transcriptomics data generated and analyzed in this study are available under Gene Expression Omnibus accession GSE300434. Code and library details for the ST analysis pipeline are available at https://github.com/alasne-uzh/migratory-astrocytes-ST. Source data are provided with this paper.

## Extended data

**Extended Data Table 1:** Clinical data of patients included in the study

Patient	B/A	Age/sex	Disease duration (years)	CNS involvement	CNS lesion studied
1	B	37/f	9	Brain, spinal cord, optic nerve	Occipital brain lesion
2	B	67/f	<1	Brain	Parietal brain lesion
3	B	21/f	<0.3	Cerebellum, brain stem, spinal cord	Cerebellar lesion
4	B	59/f	<0.3	Spinal cord	Spinal lesion
5	A	77/f	24	Brain, spinal cord, optic nerve	Periventricular brain lesion
6	A	69/f	0.5	Brain, spinal cord, optic chiasm	Periventricular brain lesion
7	A	49/f	0.3	Medulla, spinal cord	Medulla oblongata

B = biopsy, A = autopsy, f = female.

## Code availability

This paper reports an original Python code for vector-based morphological analysis that has been deposited at the Mendeley repository (10.17632/xw8fv8gt8f.1).
