# Supplementary material for: Focal astrocyte loss reveals nuclear translocation during lesion repopulation
Source: Nat Neurosci. 2026 Jul 23;29(8):1826–40. doi: 10.1038/s41593-026-02354-5 (PMC13433311; doi:10.1038/s41593-026-02354-5)
Supplement: Supplementary file 1 [file 41593_2026_2354_MOESM1_ESM.pdf]

nature neuroscience

Supplementary information

https://doi.org/10.1038/s41593-026-02354-5

Focal astrocyte loss reveals nuclear translocation during lesion repopulation

In the format provided by the authors and unedited

Supplementary Table 1

List of regeneration- and remodeling-associated genes.

Regeneration-associated gene panel	Remodeling-associated gene panel
Actn4	Actn4
Akt1s1	Brk1
Anxa7	Cotl1
Atp2b4	Emc10
Bcl2l1	Evl
C1qa	Fam107a
Cdk14	Marcks
Chst2	Pik3r2
Dact3	Rtn4
Dlg1	Sdc4
Efhd2	Sparc
Etv5	Stmn1
Fgf13	Tmsb4x
Fkbp1a	Tnr
Gfap	Tuba1a
Grn	Tubb2b
Hdac5	Usp9x
Igfbp2	Vim
Il34	
Manf	
Mast2	
Mcl1	
Ndfip1	
Pcna	
Ptprj	
Rbbp4	
Rimbp2	
Sema6b	
Smarca2	
Tmsb4x	
Trf	
Vim	
