# Supplementary material for: Focal astrocyte loss reveals nuclear translocation during lesion repopulation
Source: Nat Neurosci. 2026 Jul 23;29(8):1826–40. doi: 10.1038/s41593-026-02354-5 (PMC13433311; doi:10.1038/s41593-026-02354-5)
Supplement: Supplementary file 2 [file 41593_2026_2354_MOESM2_ESM.pdf]

nature portfolio

Corresponding author(s): Weber Bruno

Last updated by author(s): 13/05/2026

Reporting Summary

Nature Portfolio wishes to improve the reproducibility of the work that we publish. This form provides structure for consistency and transparency in reporting. For further information on Nature Portfolio policies, see our Editorial Policies and the Editorial Policy Checklist.

Statistics

For all statistical analyses, confirm that the following items are present in the figure legend, table legend, main text, or Methods section.

n/a	Confirmed	
☐	☒	The exact sample size (*n*) for each experimental group/condition, given as a discrete number and unit of measurement
☐	☒	A statement on whether measurements were taken from distinct samples or whether the same sample was measured repeatedly
☐	☒	The statistical test(s) used AND whether they are one- or two-sided<br>*Only common tests should be described solely by name; describe more complex techniques in the Methods section.*
☒	☐	A description of all covariates tested
☐	☒	A description of any assumptions or corrections, such as tests of normality and adjustment for multiple comparisons
☐	☒	A full description of the statistical parameters including central tendency (e.g. means) or other basic estimates (e.g. regression coefficient) AND variation (e.g. standard deviation) or associated estimates of uncertainty (e.g. confidence intervals)
☐	☒	For null hypothesis testing, the test statistic (e.g. *F*, *t*, *r*) with confidence intervals, effect sizes, degrees of freedom and *P* value noted<br>*Give P values as exact values whenever suitable.*
☒	☐	For Bayesian analysis, information on the choice of priors and Markov chain Monte Carlo settings
☒	☐	For hierarchical and complex designs, identification of the appropriate level for tests and full reporting of outcomes
☒	☐	Estimates of effect sizes (e.g. Cohen's *d*, Pearson's *r*), indicating how they were calculated

Our web collection on statistics for biologists contains articles on many of the points above.

Software and code

Policy information about availability of computer code

Data collection	Scanimage (version 3.8), ZEN 2011 (black edition, V7.1), MAPS software package for automatic serial section recognition and image acquisition (v 3.14, Thermo Fisher Scientific), LAS X 3.5.7.23225, Excel Microsoft (version 2018), cellSens (Olympus); 10x Genomics Visiium HD platform
Data analysis	Cellular and Hemodynamics Processing Suite (Barrett et al., 2018, Neuroinformatics) in MATLAB (R2017b; MathWorks), FIJI (ImageJ 2.1.0), Affinity Designer 2, Prism (v 10.1.1), custom written Python script (Python v 3.6.5, Mendeley repository: doi: 10.17632/xw8fv8gt8f.1) described in this paper, Li pattern recognition (Li et al., 1993) implemented in the Python scikit-image library (van der Walt et al., 2014), OpenCV (v 4.9.0); Space Ranger (v.3.1.3), R (v4.4.2) using Seurat (v5.2.1), SpotSweeper, SCTtransform and MapMyCells packages

For manuscripts utilizing custom algorithms or software that are central to the research but not yet described in published literature, software must be made available to editors and reviewers. We strongly encourage code deposition in a community repository (e.g. GitHub). See the Nature Portfolio guidelines for submitting code & software for further information.

Data

Policy information about availability of data

All manuscripts must include a data availability statement. This statement should provide the following information, where applicable:

- Accession codes, unique identifiers, or web links for publicly available datasets
- A description of any restrictions on data availability
- For clinical datasets or third party data, please ensure that the statement adheres to our policy

Authors confirm that all relevant data are included in the paper/or its supplementary information files. Further information and requests for resources should be directed to the corresponding author. This study did not generate new unique reagents. Mouse lines can be requested from the providing investigators and are protected by standard MTAs where applicable. Stained human tissue sections, whole slide scans thereof, and data related to human tissue analysis will be provided upon reasonable request. This paper reports an original Python code for vector-based morphological analysis that has been deposited at the Mendeley repository (doi: 10.17632/xw8fv8gt8f.1). Transcriptomics data generated and analyzed in this study are available under Gene Expression Omnibus accession number GSE300434. Processed data can be found at https://github.com/alasne-uzh/migratory-astrocytes-ST. Source data are provided with this paper.

Research involving human participants, their data, or biological material

Policy information about studies with human participants or human data. See also policy information about sex, gender (identity/presentation), and sexual orientation and race, ethnicity and racism.

Reporting on sex and gender	Sex and gender was not considered in the study design but sex information is reported in Extended Data Table 1.
Reporting on race, ethnicity, or other socially relevant groupings	This information has not been collected.
Population characteristics	We have not collected further information regarding population characteristics than reported in Extended Data Table 1.
Recruitment	Patient tissue with a neuropathological diagnosis of neuromyelitis optica were retrieved from the archives of the Institute of Neuropathology, University Medical Center Göttingen, Germany. Patient tissues were included if the patients were at least 18 years of age, had consented to tissue donation during surgery and to the use of their tissue for research.
Ethics oversight	Ethics committee of the University Medical Center Göttingen, Göttingen, Germany (Vote no. 19/9/10)

Note that full information on the approval of the study protocol must also be provided in the manuscript.

Field-specific reporting

Please select the one below that is the best fit for your research. If you are not sure, read the appropriate sections before making your selection.

☒ Life sciences ☐ Behavioural & social sciences ☐ Ecological, evolutionary & environmental sciences

For a reference copy of the document with all sections, see nature.com/documents/nr-reporting-summary-flat.pdf

Life sciences study design

All studies must disclose on these points even when the disclosure is negative.

Sample size	No statistical methods were used to pre-determine size of samples. Sample sizes were selected based on previous studies for similar experiments and were deemed sufficient to perform a non-clinical study.
Data exclusions	Based on pre-established exclusion criteria (based on animal protocol), animals showing signs of traumatic damage of the brain during craniotomy or bleedings were excluded from the study. Structures with insufficient signal to noise ratio during in vivo imaging were excluded. Tissue with insufficient labeling or staining was not analyzed.
Replication	All animal experiments in this study include at least three biological replicates, except for the spatial transcriptomic analysis at timepoints 5 dpi and 17 dpi (4 lesions from 2 animals, respectively). The number of replicates is mentioned for each experiment in the figure legend. The results were replicated each time.
Randomization	Randomization was not performed for this study since there was no particular intervention (e.g. treatment) planned. Whenever possible, littermate controls were used, and an equal number of mice were allocated to different groups within one cage to rule out cage effects. Analyses were performed on randomly selected datasets, as specified in the respective sections.
Blinding	Data collection and analysis were performed in a blinded manner, unless this was not possible due to an obvious disease or labeling phenotype.

Reporting for specific materials, systems and methods

We require information from authors about some types of materials, experimental systems and methods used in many studies. Here, indicate whether each material, system or method listed is relevant to your study. If you are not sure if a list item applies to your research, read the appropriate section before selecting a response.

Materials & experimental systems

n/a	Involved in the study
☐	☒ Antibodies
☒	☐ Eukaryotic cell lines
☒	☐ Palaeontology and archaeology
☐	☒ Animals and other organisms
☒	☐ Clinical data
☒	☐ Dual use research of concern
☒	☐ Plants

Methods

n/a	Involved in the study
☒	☐ ChIP-seq
☒	☐ Flow cytometry
☒	☐ MRI-based neuroimaging

Antibodies

Antibodies used

Chicken Anti-GFAP (1:1000) Abcam Cat#ab4674; RRID:AB_304558
Chicken Anti-Vimentin (1:200) Abcam Cat#ab24525; RRID:AB_778824
Mouse Anti-GFAP (1:50) Dako Cat#M0761; RRID:AB_2109952
Mouse Anti-Ki67 (MIB1; 1:200) Dako Cat#GA626; RRID:AB_2687921
Rabbit Anti-AQP4 (1:200) Sigma Aldrich Cat#A5971
Rabbit Anti-Connexin 43 (1:300) Cell Signaling 3512
Rabbit Anti-GFAP (1:1000) Dako Cat#Z0334
Rabbit Anti-Ki67 (1:500) Abcam ab16667
Rabbit Anti-Nestin (1:200) Abcam ab221660
Rabbit Anti-NeuN (1:700) Abcam ab177487
Rabbit Anti-SOX2 (1:200) Thermo Fischer Scientific PA1-094
AQP4-IgG (clone 7-5-53,0.1-3 μg/μl) Collaborator (JLB) N/A
Ctrl-IgG (clone ICOS-5-2, 1 μg/μl) Collaborator (JLB) N/A

Validation

AQP4-IgG and corresponding Ctrl-IgG were produced by Jeffrey Bennett (University of Colorado School of Medicine). Further information can be provided upon request. All other anitbodies are commercially available, widely used and validated by the manufacturer for the same application and species as used in this study. Please find details about validation in the RRID registry (https://scicrunch.org/resources/Antibodies/search?!=&q=%2A, RRID identifiers are listed above) and the manufacturer's website.

Animals and other research organisms

Policy information about studies involving animals; ARRIVE guidelines recommended for reporting animal research, and Sex and Gender in Research

Laboratory animals

C57BL/6J , Charles River , Strain code: 632
Tg(Aldh1l1-EGFP)OFC789Gsat/Mmucd MMRRC Cat#011015-UCD; RRID:MMRRC_011015-UCD
GLASTCreERT2 x Cx30/Cx43fl/fl, Own breeding
GLASTCreERT2 x ROSA26tdTomato Own breeding
GLASTCreERT2 x Ai75D (Jackson Laboratory, #025106)

Wild animals

The study did not involve wild animals.

Reporting on sex

Both sexes were used for all experiments

Field-collected samples

The study did not involve samples from the field.

Ethics oversight

All animal experiments were approved by the local veterinary authorities according to the guidelines of the Swiss Animal Protection Law, Veterinary Office, Canton Zurich (Animal Welfare Act of 16 December 2005, and Animal Protection Ordinance of 23 April 2008; license ZH217/2020) and ZH181/2024

Note that full information on the approval of the study protocol must also be provided in the manuscript.

Plants

Seed stocks	*Report on the source of all seed stocks or other plant material used. If applicable, state the seed stock centre and catalogue number. If plant specimens were collected from the field, describe the collection location, date and sampling procedures.*
Novel plant genotypes	*Describe the methods by which all novel plant genotypes were produced. This includes those generated by transgenic approaches, gene editing, chemical/radiation-based mutagenesis and hybridization. For transgenic lines, describe the transformation method, the number of independent lines analyzed and the generation upon which experiments were performed. For gene-edited lines, describe the editor used, the endogenous sequence targeted for editing, the targeting guide RNA sequence (if applicable) and how the editor was applied.*
Authentication	*Describe any authentication procedures for each seed stock used or novel genotype generated. Describe any experiments used to assess the effect of a mutation and, where applicable, how potential secondary effects (e.g. second site T-DNA insertions, mosiacism, off-target gene editing) were examined.*
